# Supplementary material for: Identification of small molecules that enhance aminoglycoside-mediated suppression of CFTR and NF1 nonsense mutations
Source: Mol Ther Oncol. 2026 Mar 10;34(2):201173. doi: 10.1016/j.omton.2026.201173 (PMC13049409; doi:10.1016/j.omton.2026.201173)
Supplement: Document S1. Figure S1 and Table S1 [file mmc1.pdf]

OMTON, Volume *34*

Supplemental information

Identification of small molecules that enhance aminoglycoside-mediated suppression of *CFTR* and *NF1* nonsense mutations

Joshua Sammons, Jianguo Chen, Kari Thrasher, Lianwu Fu, Ming Du, Hui Wen, J. Robert Bostwick, Paige Vinson, Omar Moukha-Chafiq, Corinne Augelli-Szafran, Kim M. Keeling, Deeann Wallis, Robert A. Kesterson, Steven M. Rowe, and David M. Bedwell

Table S1: qPCR Primers

Primer Name	Primer Sequence
hNF1 Forward	5’-GCTTTCGTATAAGCCCTCACAA-3’
hNF1 Reverse	5’-GCGGAATTGGTGATGATTCGATG -3’
hGUSb Forward	5’-CTGTACACGACACCCACCAC-3’
hGUSb Reverse	5’-ATTCGCCACGACTTTGTT-3’
hTBP Forward	5’-TGCACAGGAGCCAAGAGTGAA-3’
hTBP Reverse	5’-CACATCACAGCTCCCCACCA-3’

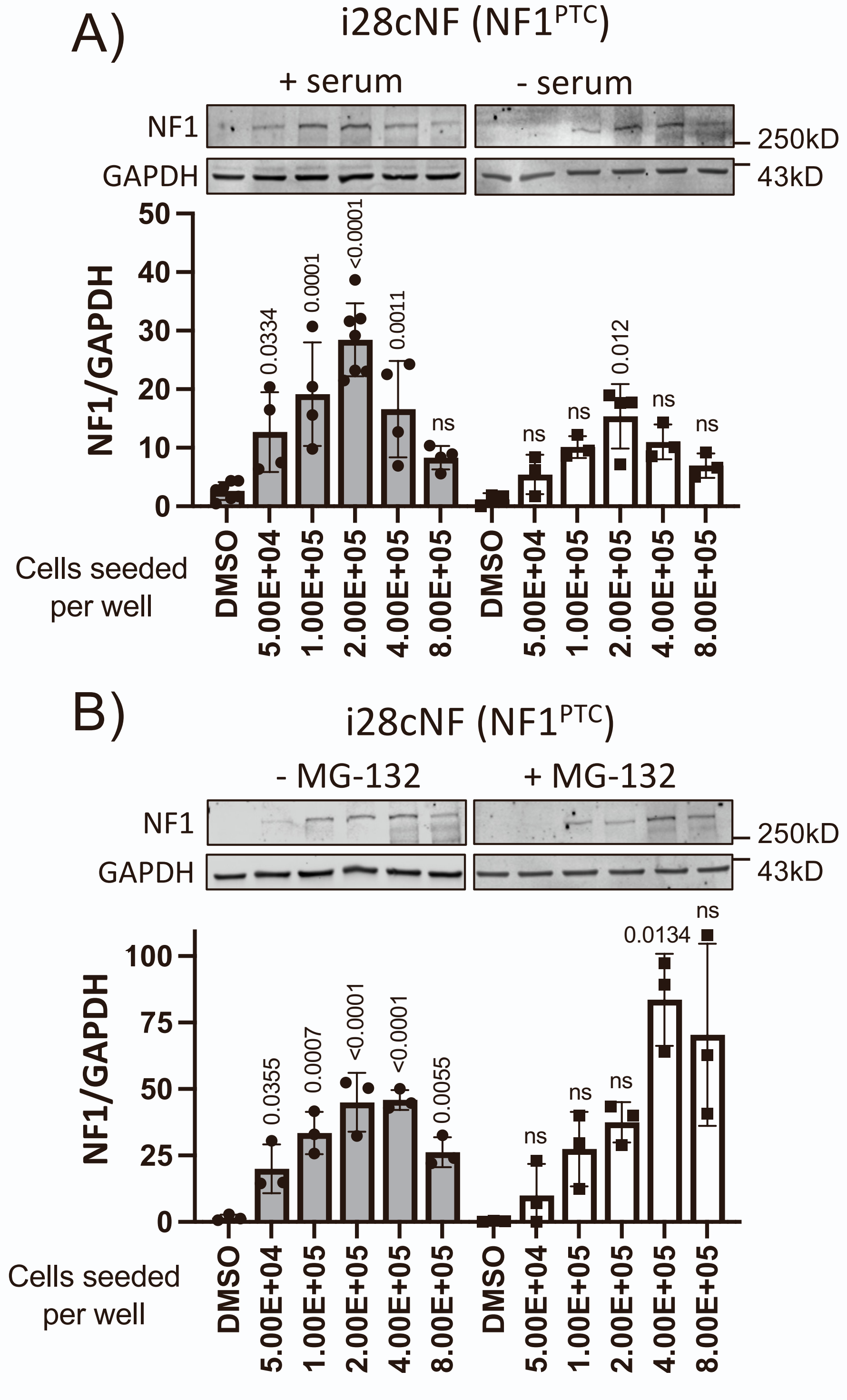

Figure S1: Optimization of neurofibromin expression in cultured NF1 Schwann cells under readthrough conditions. A) *NF1*^{PTC} (i28cNF) Schwann cells were seeded at different cell densities ($5x10^4$-$8x10^5$ cells per $9.5cm^2$ =1 well of a 6-well dish) and treated with 73.7 μM ELX-02 + 7.5 μM SRI-46124 +/- 10% FBS for 72 hours to determine the effect of confluency and serum on rescue of neurofibromin expression *via* readthrough. **B)** *NF1*^{PTC} (i28cNF) Schwann cells were seeded at different densities and treated with 73.7 μM ELX-02 + 7.5 μM SRI-46124 for 72 hours. Proteasomal inhibitor MG-132 was added during the last 24 hours to inhibit neurofibromin degradation. For A and B, DMSO treated cells were seeded at a $2x10^5$ cells per $9.5cm^2$ (1 well of a 6-well dish). For the "+ serum" and "- MG-132" cohorts, p-values (above columns) compare experimental cohorts to the respective vehicle (DMSO) control. For the "- serum" and "+ MG-132" cohorts, p-values (above columns) compare experimental cohorts to the cells lacking serum or MG-132 at the same seeding density. Data are expressed as mean (SD) and were statistically analyzed using an ordinary one-way ANOVA followed by the Dunnett's test. p values >0.05 are non-significant (ns).

Additional text related to Figure S1. Much is still not understood about how neurofibromin expression and function are regulated or how neurofibromin function intersects other signaling pathways. For example, the effect of various growth conditions on neurofibromin expression is not well-characterized. It was previously shown that multiple cell types under serum starvation express higher levels of neurofibromin than serum replete cells [1-3]. However, we report here that it can have the opposite effect on generation of full-length neurofibromin *via* PTC readthrough in Schwann cells, potentially by altering translation rates or by directly altering mechanisms related to readthrough. Further, our data suggest that sub-confluency of cultured cell cultures may be optimal for native neurofibromin expression as well as its rescue *via* readthrough. Once cells reach confluency, neurofibromin appears to be reduced in a proteasome-dependent manner.

Supplemental Material References:

1. Cichowski K, Santiago S, Jardim M, Johnson BW, Jacks T: **Dynamic regulation of the Ras pathway via proteolysis of the NF1 tumor suppressor.** *Genes Dev* **17:**449-454 (2003).
2. Gutmann DH, Cole JL, Collins FS: **Modulation of neurofibromatosis type 1 gene expression during in vitro myoblast differentiation.** *J Neurosci Res* **37:**398-405 (1994).
3. Norton KK, Mahadeo DK, Geist RT, Gutmann DH: **Expression of the neurofibromatosis 1 (NF1) gene during growth arrest.** *Neuroreport* **7:**601-604 (1996)
