# Supplementary material for: Identification of small molecules that enhance aminoglycoside-mediated suppression of CFTR and NF1 nonsense mutations
Source: Mol Ther Oncol. 2026 Mar 10;34(2):201173. doi: 10.1016/j.omton.2026.201173 (PMC13049409; doi:10.1016/j.omton.2026.201173)
Supplement: Document S2. Article plus supplemental information [file mmc2.pdf]

Molecular Therapy
Oncology
Original Article

Identification of small molecules that enhance aminoglycoside-mediated suppression of *CFTR* and *NF1* nonsense mutations

Joshua Sammons,[1,7] Jianguo Chen,[1,4,7] Kari Thrasher,[1,4,8] Lianwu Fu,[1,4] Ming Du,[1,4] Hui Wen,[4] J. Robert Bostwick,[5] Paige Vinson,[5] Omar Moukha-Chafiq,[1,4,5] Corinne Augelli-Szafran,[1,4,5] Kim M. Keeling,[1,4] Deeann Wallis,[3] Robert A. Kesterson,[3,6] Steven M. Rowe,[2,4] and David M. Bedwell[1,4]

[1]Departments of Biochemistry & Molecular Genetics, University of Alabama at Birmingham Heersink School of Medicine, Birmingham, AL 35294, USA; [2]Departments of Medicine, University of Alabama at Birmingham Heersink School of Medicine, Birmingham, AL 35294, USA; [3]Departments of Genetics, University of Alabama at Birmingham Heersink School of Medicine, Birmingham, AL 35294, USA; [4]Departments of Gregory Fleming James Cystic Fibrosis Research Center, University of Alabama at Birmingham Heersink School of Medicine, Birmingham, AL 35294, USA; [5]Southern Research, Birmingham, AL 35205, USA; [6]Pennington Biomedical Research Center, Baton Rouge, LA 70808, USA

Nonsense suppression rescues functional protein from mRNAs containing in-frame premature termination codons (PTCs). This approach employs small molecules that promote insertion of tRNAs at a PTC during translation (called "readthrough"), enabling translation to continue past the PTC to generate a full-length, functional protein. Some aminoglycosides such as G418 promote readthrough; however, their long-term use is thwarted by toxicity and modest potency. ELX-02, a novel synthetic aminoglycoside, was developed to have both enhanced readthrough activity and reduced toxicity in mammalian cells compared to traditional aminoglycosides. Recent studies suggest that at safe doses, aminoglycosides like ELX-02 may not possess sufficient efficacy to provide clinical benefits for most diseases. "Enhancer" molecules have been identified that do not possess readthrough activity themselves but enhance the efficiency of aminoglycoside-mediated readthrough. In this study, three newly identified series of enhancer molecules were tested for the ability to stimulate aminoglycoside-mediated readthrough of PTCs associated with cystic fibrosis and neurofibromatosis type 1. Although none of the enhancers alone induced readthrough, they all significantly increased readthrough via aminoglycosides at low doses (EC_{10}). Overall, our results suggest that enhancer compounds may be a viable way to overcome potency issues associated with aminoglycosides, enabling rescue of protein function from nonsense alleles while minimizing toxicity.

INTRODUCTION

Nonsense mutations are single DNA nucleotide substitutions that form an in-frame premature termination codon (PTC) in the transcribed mRNA; they comprise 11% of all known disease-causing gene loci.[1] A PTC reduces protein expression by (1) triggering nonsense-mediated mRNA decay (NMD) to prevent mRNAs from being translated and (2) prematurely terminating translation of mRNAs that escape NMD. These PTC-directed mechanisms result in the production of truncated proteins that are typically non-functional and/or unstable. Because nonsense mutations generally lead to negligible protein function, they are often associated with the most severe clinical presentations of a disease. In addition, by severely reducing both mRNA and protein levels, a nonsense mutation limits the feasibility of many therapeutic strategies. Nonsense suppression therapy is one approach being explored to rescue functional protein from nonsense alleles. This therapeutic strategy uses small molecules that target the translational machinery to suppress translation termination at PTCs, which can both rescue partial expression of full-length, functional protein and antagonize NMD.[2–5]

In eukaryotes, translation termination occurs when eukaryotic release factor 1 (eRF1) recognizes a stop codon (UAA, UAG, or UGA) in the ribosomal acceptor (A) site. eRF1, assisted by its GTPase-binding partner, eRF3, induces hydrolysis of the peptidyl-tRNA ester bond to release the nascent protein.[6] At a low frequency, aminoacyl tRNAs that can Watson-Crick base-pair with two of the three nucleotides of a PTC (known as near-cognate tRNAs) compete with eRF1 for binding to PTCs in the A site, leading to the insertion of an amino acid into the nascent polypeptide at the PTC position. This mechanism, referred to as "readthrough," allows translation elongation to continue downstream of the PTC in the correct

Received 8 October 2025; accepted 5 March 2026;
https://doi.org/10.1016/j.omton.2026.201173.

[7]These authors contributed equally to the current study.

[8]Present address: University of Rochester School of Medicine and Dentistry, Rochester, NY 14642, USA

Correspondence: David M. Bedwell, Departments of Biochemistry & Molecular Genetics, University of Alabama at Birmingham Heersink School of Medicine, Birmingham, AL 35294, USA.
E-mail: dbedwell@uab.edu

© 2026 The Author(s). Published by Elsevier Inc. on behalf of The American Society of Gene and Cell Therapy. This is an open access article under the CC BY license (http://creativecommons.org/licenses/by/4.0/).

ribosomal reading frame such that a full-length protein is generated. Readthrough compounds act by increasing the frequency of near-cognate tRNA accommodation at PTCs. Due to differences in the local messenger ribonucleoprotein (mRNP) structure,[7] normal stop codons are less susceptible to readthrough than PTCs, creating a therapeutic window for readthrough compound action to produce functional protein from nonsense alleles via termination suppression.

Several mechanistic classes of small molecules have been identified that mediate PTC readthrough in mammalian cells.[8–10] However, three recurring limitations have been noted. These have (1) poor efficacy (the maximal readthrough obtained), (2) poor potency (the concentration of a compound needed for a response), and (3) off-target effects (that can result in toxicity). Individually or collectively, these problems have hindered the utility of readthrough agents as long-term therapeutics. Aminoglycosides are the best studied readthrough agents, which were first demonstrated to suppress disease-associated PTCs in the 1990s.[11,12] Major obstacles that prohibit the long-term clinical use of aminoglycosides include both their poor potency and off-target effects, which can give rise to nephrotoxicity and ototoxicity.[13] However, newer synthetic aminoglycosides such as ELX-02 have been designed to more efficiently target cytoplasmic ribosomes, which increases their readthrough efficiency and reduces their toxicity.[14–16] While phase 1 clinical trials confirmed the safety of ELX-02,[17,18] subsequent efficacy studies suggest that, at safe doses, ELX-02 does not rescue enough protein function to impart therapeutic benefits for genetic diseases with a high threshold for correction.[19] However, aminoglycoside readthrough efficiency can be enhanced by the co-administration of other mechanistic classes of readthrough agents, with some conditions generating additive or, in some cases, synergistic increases in readthrough with relatively low aminoglycoside concentrations.[2,20–24] In addition, "enhancer" compounds have been identified which possess little or no readthrough activity themselves but augment the readthrough activity of aminoglycosides, enabling more efficient readthrough at lower aminoglycoside doses.[20,25–28]

Cystic fibrosis (CF) is caused by the loss of functional cystic fibrosis transmembrane conductance regulator (CFTR) protein, which serves as a cAMP-activated chloride channel that regulates the movement of chloride and bicarbonate ions across epithelia in the lungs, pancreas, and intestines.[29] Progressive decline in lung function is the main cause of morbidity and mortality among CF patients. Loss of CFTR function leads to accumulation of thick mucus on the apical surface of epithelia, which reduces mucociliary transport and leads to recurrent infections and chronic inflammation. However, CFTR is also thought to play a significant tumor-suppressor role in colorectal cancer.[30,31] Roughly 7% of CF patients carry a nonsense mutation.[32]

Neurofibromatosis type 1 (NF1) is caused by loss of functional neurofibromin, a tumor suppressor that functions as a GTPase-activating protein (GAP) that promotes the conversion of active Ras-GTP to inactive Ras-GDP, thus downregulating the Ras signaling cascade.[33] Insufficient neurofibromin function leads to constitutive Ras activity and increased phosphorylation of downstream MAPKs, including the serine/threonine kinases MEK and ERK1/2. This results in unchecked cell growth that begets formation of benign peripheral nerve tumors known as neurofibromas. Other common NF1 symptoms include café-au-lait spots, Lisch nodules, learning deficiencies, and autism spectrum disorder. Roughly 20% of NF1 patients harbor a germline nonsense mutation on at least one allele of the *NF1* gene.[34]

In the current study, we carried out a high throughput screen (HTS) to identify new small molecules that enhance the ability of aminoglycosides to suppress termination at PTCs. Hits from the screen were validated by testing their ability to enhance aminoglycoside-mediated readthrough of nonsense mutations associated with CF and NF1. We found that the novel enhancer molecules identified in this study allowed aminoglycosides to suppress PTCs in both the *CFTR* and *NF1* genes at much lower concentrations than were required for efficient readthrough with aminoglycosides alone. Overall, our data suggest that aminoglycoside enhancers may be a feasible way to improve aminoglycoside potency such that lower, nontoxic aminoglycoside dosages can be used to promote efficient readthrough. The use of aminoglycoside enhancers could potentially spur the development of clinically relevant nonsense suppression treatments.

RESULTS

Identification of new compounds that enhance aminoglycoside-mediated readthrough

We initially performed HTS of a diverse library of 532,062 low molecular weight compounds to identify new chemical series that enhance the readthrough of PTCs by aminoglycosides. Because previous studies found that readthrough-enhancing small molecules could be identified that generate little or no readthrough activity alone,[25,26] we performed two parallel HTS; one with the library alone and the second with the same library in the presence of a low dose (EC_{20}) of the aminoglycoside, G418. To perform the HTS, we utilized a previously described reporter that contains a PTC at codon W134 (TG<u>G</u>→TG<u>A</u>) in the NanoLuc luciferase (NanoLuc^{W134X}) that inactivates NanoLuc activity (Figure 1A).[2] In this reporter, the NanoLuc cDNA is fused with an in-frame downstream beta-globin gene containing intronic sequences that elicit NMD of the reporter mRNA. In this way, the NanoLuc^{W134X} reporter (1) mimics the effect of disease-related PTCs on mRNA abundance and (2) can be used to identify compounds that not only promote readthrough but also inhibit NMD. High levels of readthrough can antagonize NMD,[2–5] and thus, effective readthrough-enhancing compounds can amplify the overall NanoLuc signal by mediating readthrough as well as by inhibiting NMD. This reporter was stably transfected into the immortalized, wild-type (WT) human bronchial epithelial 16HBE14o- (16HBE) cell line. A monoclonal 16HBE cell line with the NanoLuc reporter integrated into the genome was subsequently derived and used to perform the HTS. A single concentration of each

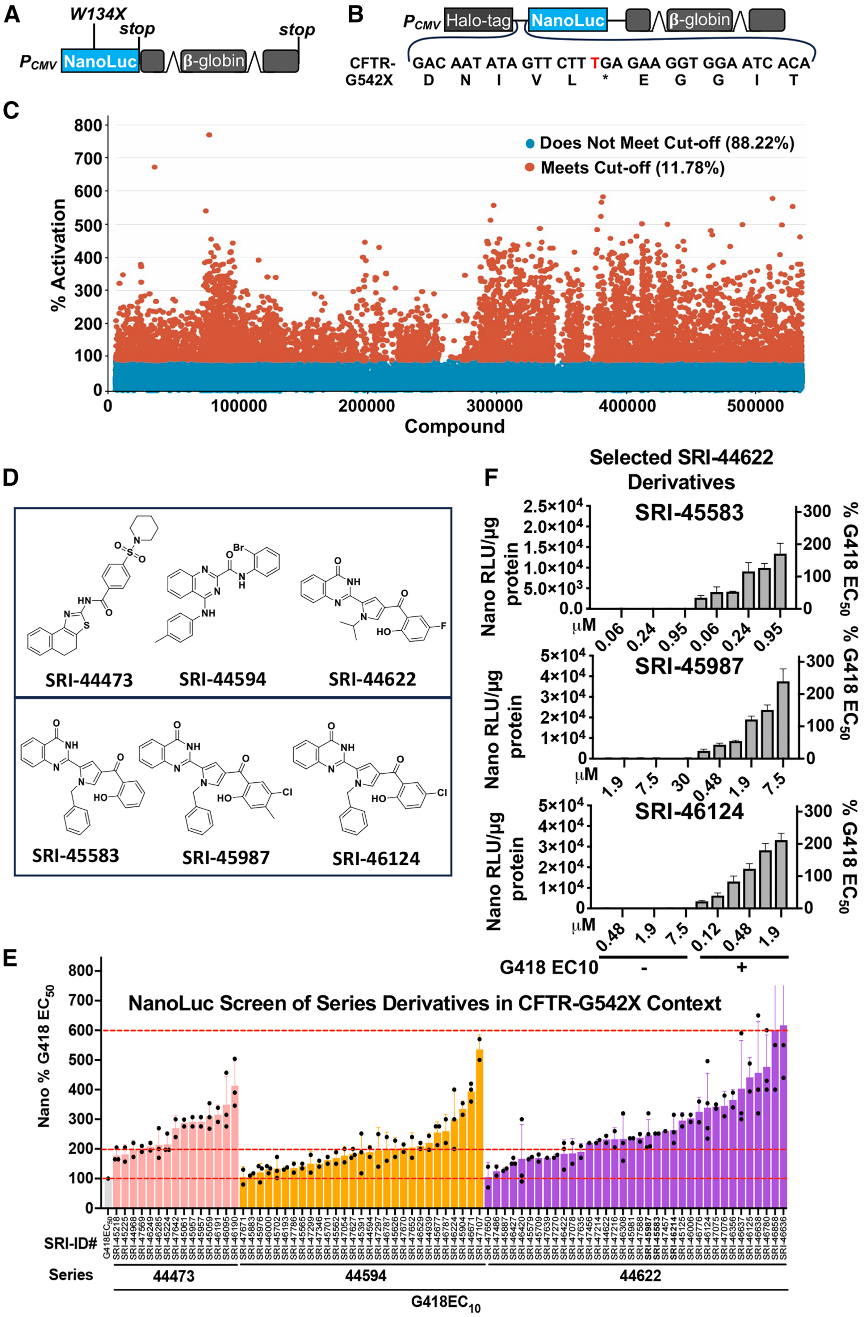

Figure 1. Identification of new aminoglycoside enhancer compounds

NanoLuc readthrough reporters expressed in wild-type (WT) 16HBE14o- cells were generated using (A) NanoLuc W134X to perform high-throughput screens (HTS) and (B) NanoLuc *CFTR* G542X to validate HTS hits. (C) NanoLuc activity (% activation) (via the NanoLuc W134X reporter cells; see (A) in response to treatment with 30 μM of compound alone or co-administered with G418 EC_{20} (100 μg/mL); untreated cells = 0% activation; cells treated with G418 EC_{20} = 100% activation. (D) Structures of select HTS hits. (E) Mean (SD) NanoLuc activity generated from *CFTR* G542X readthrough reporter cells (see B) treated with derivatives from three different chemical series of enhancers in the presence of G418 EC_{10}. (F) Mean (SD) NanoLuc activity generated from *CFTR* G542X readthrough reporter cells (see B) treated with different doses of key enhancer molecules in the absence and presence of G418 EC_{10}. The activity obtained with the maximum dose for each compound without significant cell loss is shown on the *x* axis.

compound (30 μM) was tested, and activation was calculated in compound-treated wells by setting the average NanoLuc response of the cell-only control as 0% activation and the average NanoLuc response at the G418 EC_{20} (100 μg/mL) control as 100% activation (Figure 1C). The assay performed well with an average Z' score = 0.76. No plates with Z' <0.5 were used for hit selection. The top 5,723 compounds with an activation >100% in wells containing G418 EC_{20} were selected as hits for confirmation using secondary screening and cytotoxicity testing. A total of 853 compounds had maximum activation values >100%, and 628 of these had measurable potency values. Activities of these confirmed hits were compared across multiple assays to select compounds for further validation.

Enhancers combined with G418 rescue CFTR function

Among the hits discovered from the HTS, three different chemical scaffolds (SRI-44473, SRI-44594, and SRI-44622) (Figure 1D, top) were identified as promising candidates. Series of derivatives from each scaffold were subsequently synthesized and assayed using a second NanoLuc reporter that carries the CF-associated *CFTR* G542X context, consisting of the PTC (GGA→TGA) flanked by five codons of natural upstream and downstream *CFTR* mRNA sequence (Figure 1B). We transfected this *CFTR*-G542X NanoLuc reporter into *CFTR* WT 16HBE cells[35] and generated a stable, monoclonal cell line. These cells were then used to examine the ability of candidates from these enhancer series to promote readthrough of the CF-relevant G542X PTC.

We tested 18 derivatives from the SRI-44473 series, 42 from the SRI-44594 series, and 43 from the SRI-44622 series. We found that none of the derivatives alone could stimulate appreciable readthrough of the *CFTR* G542X context (data not shown). However, many of these derivatives enhanced G418-mediated readthrough (Figure 1E). Because the SRI-44622 series contained derivatives that mediated

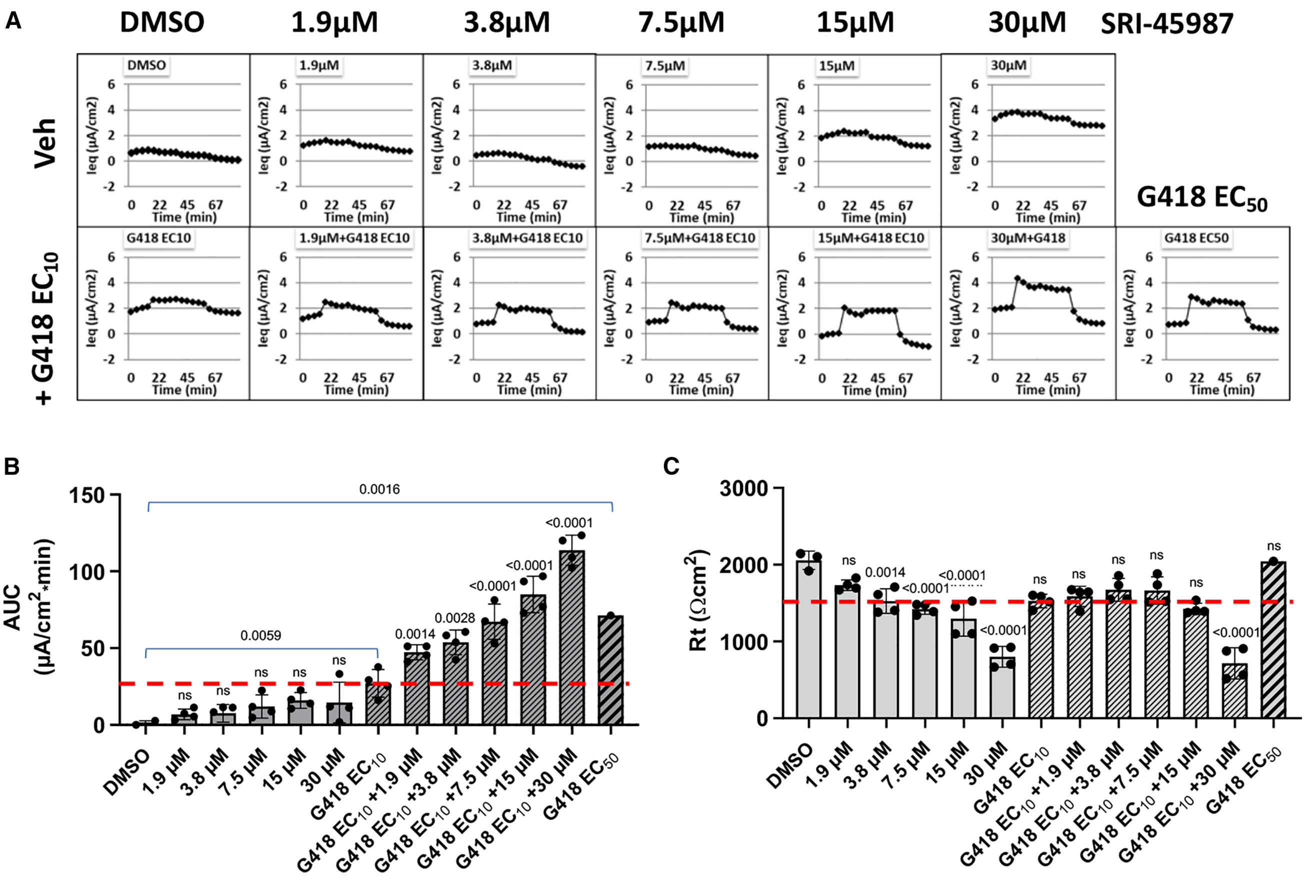

Figure 2. Enhancer compound SRI-45987 rescues CFTR channel function in *CFTR* G542X 16HBEs

Cells were treated with SRI-45987 (0–30 μM) +/− G418 EC_{10} (6.53 μM) for 72 h. CFTR activity was assessed using the TECC assay. (A) Representative equivalent current (Ieq) tracings from treatments with SRI-49587 +/− G418 EC_{10}. (B) The corresponding summary of CFTR activity, calculated as the mean (SD) area under the curve (AUC) between forskolin-induced (10 μM) stimulation of CFTR activity and inhibition of CFTR with CFTRinh-172 (20 μM) (n = 1–4 per group). (C) Mean (SD) cell resistance values in all treatment groups, measured by the TECC assay before the addition of forskolin. Dashed red lines indicate the values of AUC (in B) and Rt (in C) observed in the presence of G418 EC_{10} alone. Data are expressed as mean (SD) and were statistically analyzed using an ordinary one-way ANOVA followed by Holm-Sidak's test. In (B), p values are calculated to compare cohorts treated with G418 EC_{10} or G418 EC_{50} (brackets) or with SRI-45987 alone (above each column) with the DMSO control. p values were also calculated to compare cells treated with G418 EC_{10} alone to those co-treated with G418 EC10 + SRI-45987 (right side of graph above hatched columns). In (C), p values were calculated comparing all treated samples to the DMSO control. p values > 0.05 are non-significant (ns).

significant readthrough enhancement with the least cellular toxicity, we chose to further test three of the most promising derivatives from this series (SRI-45583, SRI-45987, and SRI-46124) (Figure 1D, bottom). We treated the *CFTR* G542X NanoLuc 16HBE reporter cells with each compound alone or co-administered with G418 EC_{10} (Figure 1F). We found that these three analogs enhanced G418 activity with similar efficacy when expressed as a percent of the G418 EC_{50} value (171%–239% of G418 EC_{50}). The maximum concentration used for each compound was chosen that provided optimal readthrough activity without significant cell toxicity. These maximal readthrough values obtained represented a 5- to 10-fold increase in total readthrough activity when compared to readthrough mediated by G418 EC_{10} alone.

We next tested each of these compounds for the ability to enhance G418-mediated readthrough and restore CFTR activity in a gene-edited 16HBE14o- cell line carrying the G542X nonsense mutation (GGA→TGA) in the endogenous *CFTR* locus.[35] In preliminary experiments, we found that SRI-45987 restored the most CFTR function with the least toxicity in the *CFTR* G542X 16HBE cell line, so this compound was tested more extensively. *CFTR* G542X 16HBE cells were treated with different concentrations of SRI-45987+/− G418 EC_{10}. CFTR chloride channel function was then measured using transepithelial chloride conductance (TECC) assays. Representative raw equivalent current (Ieq) traces are shown in Figure 2A, and the composite data of multiple experiments are expressed as area under the curve (AUC) (Figure 2B). We saw only a minimal increase in

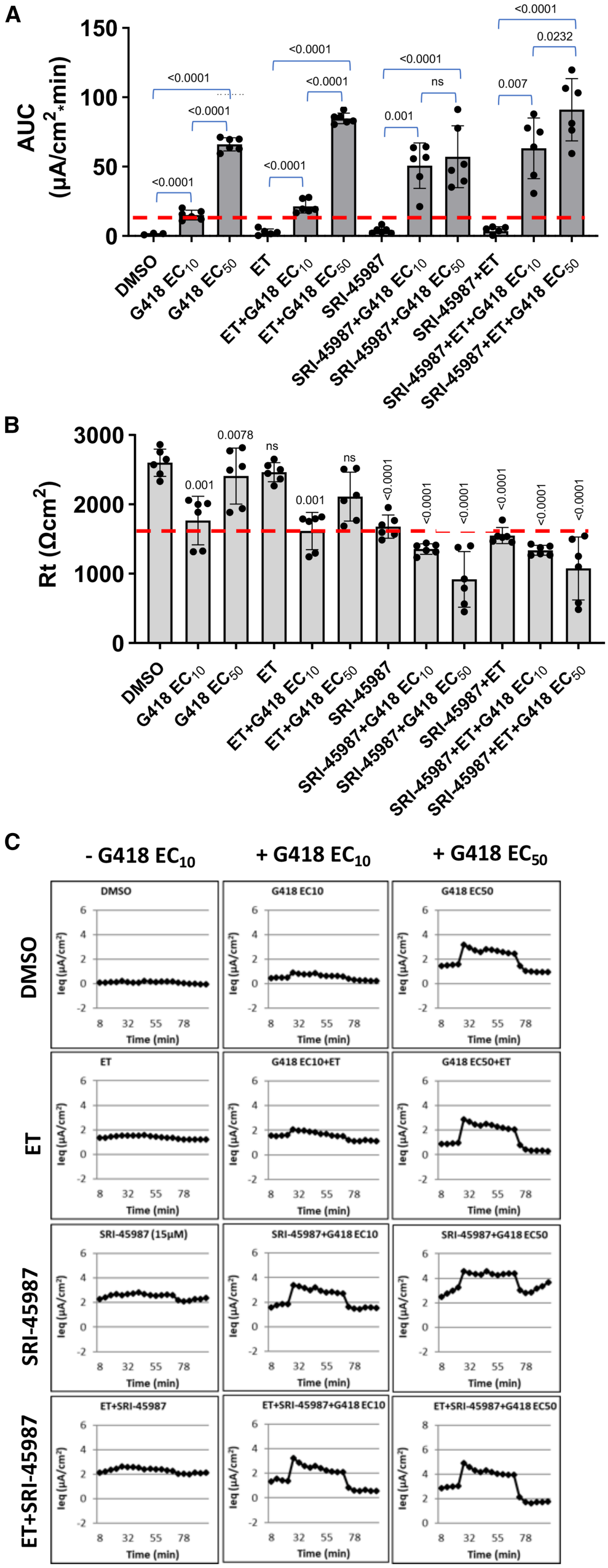

Figure 3. Enhancer compound SRI-45987 enhances readthrough mediated by low G418 doses

CFTR G542X 16HBEge cells were treated for 72 h with DMSO, G418 EC_{10} (6.53 μM) or G418 EC_{50} (39.2 μM), CFTR correctors ET (3 μM each), or SRI-45987 (15 μM) alone or in various combinations as indicated. TECC assays were then conducted with the acute addition of forskolin, VX-770, and CFTRinh-172. (A) CFTR activity was calculated as the meand (SD) area under the curve (AUC) between the forskolin-induced (10 μM) stimulation of CFTR activity and the inhibition of CFTR with CFTRinh-172 (20 μM) ($n = 6$ per group). (B) Mean (SD) cell resistance values for all treatment groups were measured prior to the addition of forskolin. (C) Representative equivalent current (Ieq) tracings for all treatment groups. Dashed red lines indicate the values of AUC (in A) and Rt (in B) observed in the presence of G418 EC_{10} alone. Data are expressed as mean (SD) and were statistically analyzed using an ordinary one-way ANOVA followed by Holm-Sidak's test. In (A), p values comparing different cohorts are indicated by brackets. In (B), p values are calculated that compare the treated samples to the DMSO control. p values > 0.05 are non-significant (ns).

CFTR function with SRI-45987 alone, but the compound provided a strong dose-dependent increase in CFTR conductance when combined with G418 EC_{10}. The cellular resistance (Rt) is a proxy for cell monolayer integrity (Figure 2C). While CFTR activity showed a dose-dependent increase up to 30 μM SRI-45987, a dose of 15 μM gave the best AUC without a large decrease in resistance. Note that the TECC assay is commonly expressed as AUC of the trace in Figure 2A. AUC is a direct measure of CFTR activity in the cell monolayer and is based on forskolin-stimulated and CFTR-Inh-172 inhibited current. In contrast, Rt (total resistance) represents overall epithelial barrier integrity. This value can reflect the integrity of tight junctions between cells but could also have a component based on the contribution of CFTR-mediated chloride transport to that resistance. In general, when the Rt value is reduced without corresponding CFTR activity, we interpret these as an indicator of reduced integrity of the cell monolayer and tight junctions. In this case, we interpret the reduced Rt in the presence of 30 μM SRI-45987 (whether with or without G418) is due to a reduction in integrity of the cell monolayer/tight junctions.

Previous studies found that suppression of *CFTR* PTCs frequently results in a mixture of amino acids inserted at the site of the PTC.[36–38] In some cases, this leads to reduced CFTR expression/function, but we found that the addition of CFTR correctors, which promote CFTR protein folding and localization to the apical membrane, frequently attenuates that reduction.[39] To examine the effect of CFTR correctors, we treated *CFTR* G542X 16HBE cells with combinations of 15 μM SRI-45987, G418 EC_{10} or EC_{50}, and two small molecule CFTR correctors: elexacaftor (E) and tezacaftor (T). These correctors improve CFTR processing in the ER and enhance cellular trafficking, allowing more CFTR protein to reach the cell surface. This often enhances the amount of CFTR activity measured, since CFTR must be at the cell surface to promote chloride transport across the plasma membrane. An FDA-approved drug that includes ET, along with the CFTR potentiator ivacaftor (I) is marketed as Trikafta to treat CF. The level of CFTR function (AUC) measured under each condition is shown in Figure 3A. We found that treatment with

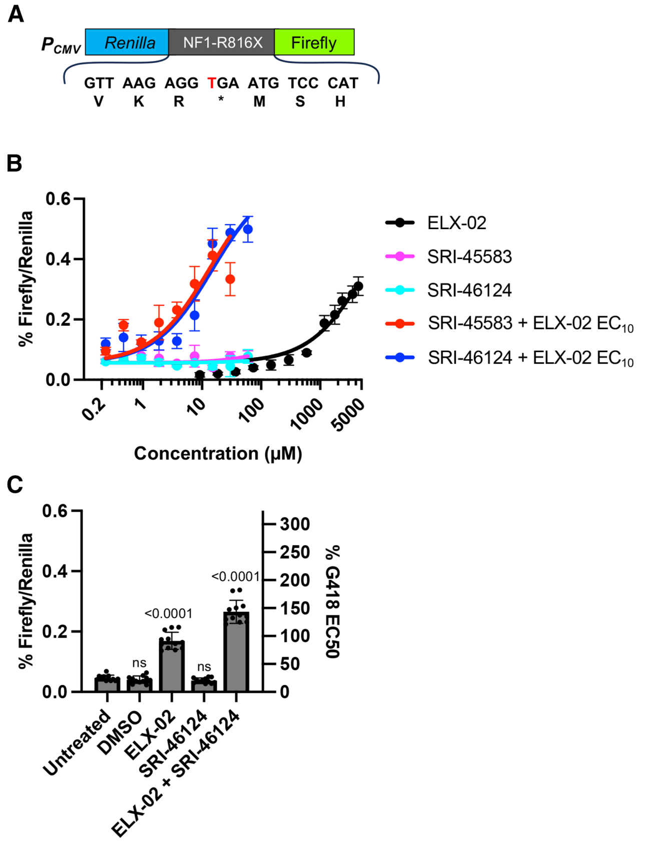

Figure 4. Enhancer compounds increase ELX-02 mediated readthrough of the *NF1* R816X context

(A) Dual luciferase readthrough (RT) reporter carrying a naturally occurring, NF1-associated nonsense mutation R816X ($NF1^{R816X}$) between the two luciferases. The inserted R816X context includes the UGA premature termination codon (PTC) flanked by three codons of natural upstream and downstream *NF1* mRNA context. (B) Dose-response curves for ELX-02 (EC_{10} = 73.7 μM) or for SRI-46124 and SRI-45583 +/− ELX-02 EC_{10} were generated in a WT Schwann cell line stably expressing the *NF1* R186X dual luciferase RT reporter. (C) *NF1* R816X RT assays in Schwann cells treated with 0.3% DMSO (vehicle), 1.35 mM ELX-02 (EC_{50}), and 7.5 μM SRI-46124. Data are expressed as mean (SD) and were statistically analyzed using an ordinary one-way ANOVA followed by Dunnett's multiple comparisons test. *p* values were calculated to compare ELX-02-treated cells to the untreated control. *p* values < 0.0001 when ELX-02 + SRI-46124 is compared to the untreated or the ELX-02 alone controls. *p* values > 0.05 are non-significant (ns).

G418 EC_{50} rescued a significant amount of chloride transport in *CFTR* G542X 16HBE cells (Figure 3A), but as expected, a much more modest response was observed at G418 EC_{10}. The addition of ET alone, SRI-45987 alone, or a combination of the two provided no enhancement of chloride transport above the vehicle control. Combining SRI-45987 with G418 EC_{10} provided a strong increase in CFTR function compared to the same dose of G418 alone (indicated by the dotted red line). In contrast, CFTR function in cells treated with SRI-45987 with G418 EC_{50} was not significantly different from G418 EC_{50} alone, indicating that readthrough efficiency was not increased by the enhancer at high G418 concentrations. Instead, these results show that the addition of G418 EC_{10} plus enhancer greatly reduced the amount of G418 required to significantly increase readthrough activity, indicating that the enhancer acts to increase aminoglycoside potency. The addition of ET to the G418/SRI-45987 combination offered only a modest further increase when compared to the G418/enhancer combination only. We were unable to consistently correlate CFTR function with rescue of full-length CFTR protein expression by western blot (data not shown), suggesting that the level of CFTR expression restored was at the lower limit of detection. The cellular resistance (Figure 3B) was modestly reduced when G418 EC_{10} was combined with SRI-45987, and more significantly reduced when a G418 EC_{50}/enhancer combination was used. Representative raw equivalent current (Ieq) traces from which these data were derived are shown in Figure 3C.

Enhancers augment aminoglycoside-mediated readthrough of PTCs associated with NF1

To examine the broader utility of these aminoglycoside enhancers, we next tested whether they also augment readthrough of nonsense mutations in a human NF1 cell model. Since neurofibromas often develop from Schwann cells,[40] they represent a cell type that is highly relevant to NF1.

To initially explore readthrough of NF1-associated nonsense mutations in Schwann cells, we constructed a dual luciferase reporter containing the *NF1* R816X PTC (CGA→TGA) flanked by three codons upstream and downstream of natural *NF1* sequence (Figure 4A). Such a minimal sequence context has been shown to accurately reflect the overall readthrough associated with full-length contexts.[41] This reporter construct was transfected into the ipn02.8 immortalized WT human Schwann cell line, stable transfectants were selected, and a monoclonal cell line was generated.

Initial experiments with G418 indicated positive results at low concentrations, but not at higher doses, which we hypothesized was due to cellular toxicity (data not shown). Therefore, we switched to ELX-02, a synthetic aminoglycoside that induces readthrough by the same mechanism as G418[42,43] to examine readthrough in the reporter Schwann cells. We chose ELX-02 because it exhibits less toxicity than G418 and has been used in recent clinical trials[19] (ClinicalTrials.gov identifiers NCT04069260 and NCT05448755). Since SRI-45987 was tested in the CF model as described previously, we tested the other two aminoglycoside enhancers from Figure 1F (SRI-45583 and SRI-46124) for their ability to enhance suppression of the *NF1* R816X PTC context. We first established ELX-02 dose-response curves in the reporter Schwann cells. Cells treated with an ELX-02 EC_{10} (74 μM) for 72 h were then used to determine the dose response for each enhancer. We found that both enhancer compounds increased the readthrough potency of ELX-02 at its EC_{10} by >60-fold in the reporter Schwann cells (Figure 4B). Based on these results and the lower toxicity of SRI-46124, we then examined the effect of the enhancers on the overall efficacy of ELX-02 at its EC_{50}. Figure 4C shows that while SRI-46124 alone did not induce readthrough, the addition of a high ELX-02 EC_{50} dose combined with

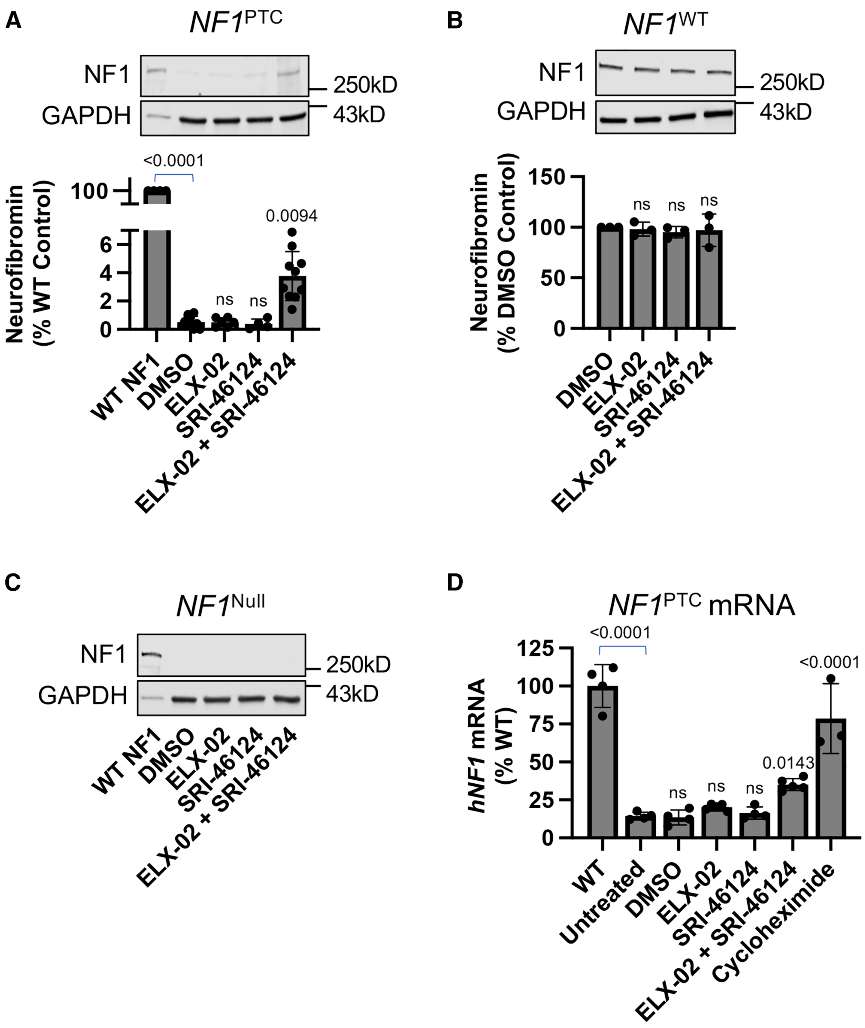

Figure 5. Enhancer SRI-46124 increases the level of full-length, endogenous neurofibromin rescued by ELX-02 mediated readthrough

Western blots and quantitation of neurofibromin in (A) *NF1*^PTC^, (B) *NF1*^WT^, and (C) *NF1*^null^ Schwann cell lines. Schwann cells treated for 72 h with the vehicle control (0.3% DMSO), 73.7 μM ELX-02, or 7.5 μM SRI-46124, alone and in combination. (D) Quantitation of human *NF1* mRNA levels via quantitative reverse-transcription PCR (RT-qPCR), with *hGUSb* and *hTBP* used as reference genes in *NF1*^WT^ or *NF1*^PTC^ Schwann cells treated with 0.3% DMSO, 73.7 μM ELX-02, or 7.5 μM SRI-46124 for 72 h, or with 10 μg/mL of the NMD inhibitor cycloheximide for 24 h. Data are expressed as mean (SD) and were statistically analyzed using an ordinary one-way ANOVA followed by Dunnett's multiple comparisons test. *p* values compare experimental cohorts to the untreated or vehicle (DMSO) control. *p* values > 0.05 are non-significant (ns).

SRI-46124 induced more readthrough of the *NF1*-R816X PTC than ELX-02 alone, but the effect was <2-fold. When taken together, these results show that the enhancer SRI-46124 improves the ELX-02 readthrough potency much more than its efficacy for the *NF1*-R816X context in reporter Schwann cells.

Treatment with both ELX-02 and SRI-46124 restores expression of full-length neurofibromin in Schwann cells

We next examined the effect of SRI-46124 on ELX-02-mediated readthrough in a human Schwann cell line that carries nonsense mutations in the endogenous *NF1* locus. In these experiments, we used the i28cNF immortalized Schwann cell line that carries TGA nonsense mutations (S1053X/S1078X) on both *NF1* alleles ($NF1^{PTC}$).[44] Since NF1 expression and abundance were previously reported to vary as a function of culture confluency,[45,46] we initially carried out readthrough assays with the i28cNF immortalized Schwann cell line to determine the optimal seeding density. Our results confirmed that the abundance of NF1 protein following readthrough decreased at high cell confluency in a proteasome-dependent manner (Figure S1). The optimal conditions observed (a seeding density of 2×10^5 cells) were used in subsequent experiments.

Cells were treated with ELX-02 alone or ELX-02 combined with SRI-46124 for 72 h, and western blotting was then performed to determine whether full-length neurofibromin could be detected. Full-length protein was not observed after treatment with either ELX-02 or SRI-46124 alone (Figure 5A). However, following treatment with both ELX-02 + SRI-46124, we observed rescue of full-length neurofibromin (estimated to be 4%–5% of the level of protein observed in $NF1^{WT}$ Schwann cells [Figure 5A]; note that 5% of $NF1^{WT}$ lysate compared to $NF1^{PTC}$ lysate was used as a loading control). In contrast, increases in neurofibromin were not observed in $NF1^{WT}$ Schwann cells (Figure 5B) or in the ipNF95.11b C NF1 Schwann cell line that carries non-PTC null mutations ($NF1^{Null}$) (Figure 5C).

The presence of a PTC in an mRNA often triggers NMD, which reduces steady-state mRNA abundance.[47] However, it has been previously shown that efficient readthrough of PTCs with small molecules can correspondingly restore the abundance of the PTC-containing mRNA.[2–5] Similarly, the use of anticodon engineered (ACE)-tRNAs to suppress termination at PTCs can also result in an increase of PTC-containing *CFTR* mRNA, suggesting that PTC suppression can antagonize NMD-mediated mRNA destabilization.[48] Based on these observations, we next sought to determine whether readthrough conditions that promote increased expression of full-length neurofibromin in $NF1^{PTC}$ Schwann cells could also increase *NF1*

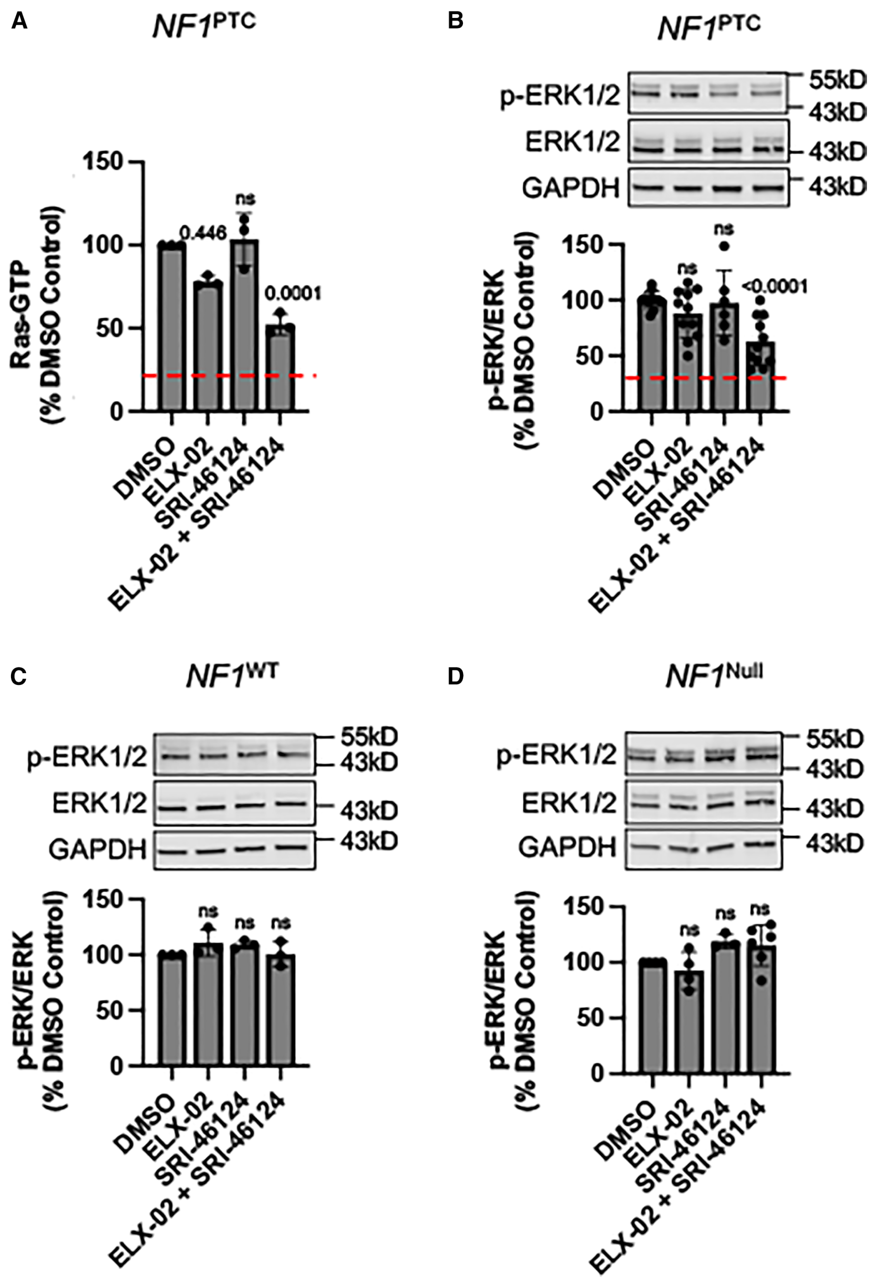

Figure 6. Enhancer SRI-46124 increases the level of neurofibromin function rescued by ELX-02-mediated readthrough

(A) Ras-GTP levels via ELISA in lysates from *NF1*^{PTC} Schwann cells treated with 73.7 μM ELX-02 (EC_{10}) or 7.5 μM SRI-46124 alone or combined. Western blots of phosphorylated-ERK (pERK1/2) and total-ERK (ERK1/2) in (B) *NF1*^{PTC}, C) *NF1*^{WT}, and D) *NF1*^{null} Schwann cell lines treated with 73.7 μM ELX-02 or 7.5 μM SRI-46124 alone or combined. Data are expressed as mean (SD) and were statistically analyzed using an ordinary one-way ANOVA followed by Turkey's multiple comparisons test. *p* values compare experimental cohorts with the vehicle (DMSO) control. *p* values > 0.05 are non-significant (ns). The red dashed line in (A) represents the level of Ras-GTP in WT cells, while the red dashed line in (B) represents the level of pERK/ERK in WT cells.

mRNA abundance. Using qPCR, we found that the combination of ELX-02 and SRI-46124 significantly increased the steady-state abundance of *NF1* mRNA 2-fold compared to the untreated and DMSO controls, reaching ~25% of the *NF1*^{WT} control (Figure 5D). For comparison, the strong NMD inhibitor cycloheximide raised the *NF1*^{PTC} mRNA abundance to ~75% of the *NF1*^{WT} control cells. Taken together, these data suggest that the level of readthrough reached by co-treatment with ELX-02 and SRI-46124 attenuated NMD of PTC-containing *NF1* mRNAs in human Schwann cells.

Restoration of full-length neurofibromin via PTC readthrough reduces Ras-GTP and the level of ERK phosphorylation in NF1 Schwann cells

Loss of neurofibromin gives rise to elevated Ras-GTP levels and enhanced Ras signaling, subsequently leading to increased cell proliferation and neurofibroma formation.[49,50] The results presented previously suggest that the combination of ELX-02 and SRI-46124 promotes readthrough of *NF1* PTCs, resulting in rescue of full-length neurofibromin. To determine whether the neurofibromin rescued by readthrough possessed Ras-GAP activity, we examined Ras-GTP levels using an ELISA-based assay. As expected, we found that untreated *NF1*^{PTC} Schwann cells contained 4-fold more Ras-GTP than *NF1*^{WT} Schwann cells (Figure 6A; red dashed line represents *NF1*^{WT} levels). Treatment of these cells with ELX-02 alone caused a modest (~20%) decrease in Ras-GTP in the *NF1*^{PTC} cell line, while SRI-46124 alone had no effect of Ras-GTP levels. However, treatment with both ELX-02 + SRI-46124 reduced Ras-GTP levels by ~50% (Figure 6A). These results are consistent with an enhancement of ELX-02-mediated readthrough by SRI-46124. While the Ras-GTP level did not reach WT levels, this reduction may be sufficient to provide at least a partial therapeutic benefit.

Because Ras activation leads to phosphorylation of downstream effectors such as ERK1/2 (*p*-ERK),[51,52] we next tested whether ELX-02 + SRI-46124 could reduce the level of *p*-ERK relative to total-ERK in the *NF1*^{PTC} Schwann cell line. Consistent with constitutive Ras activation in cells lacking neurofibromin, untreated *NF1*^{PTC} Schwann cells had increased *p*-ERK/total-ERK levels that were ~4-

fold higher than the *NF1*^{WT} cell line (indicated by the red dotted line) (Figure 6B). After 72 h of treatment, ELX-02 treatment alone resulted in a slight decrease in *p*-ERK, but this trend did not reach statistical significance. However, treatment of these cells with ELX-02 + SRI-46124 resulted in a significant 2-fold decrease in *p*-ERK compared to the DMSO control. While promising, it is not yet known if this level of reduction is sufficient to provide a therapeutic benefit in NF patients. In contrast, treatment with either compound alone or in combination had no effect on *p*-ERK in *NF1*^{WT} (Figure 6C) or *NF1*^{null} (Figure 6D) Schwann cell lines. These results indicate that the changes seen in *p*-ERK levels were dependent upon readthrough of the *NF1*^{PTC} allele and restoration of full-length, functional neurofibromin.

DISCUSSION

PTCs are frequently found in tumor suppressor genes. Nonsense suppression agents have been shown to rescue protein function from several tumor suppressor nonsense variants, including *TP53*, *APC*, *ATM*, and *PTEN*.[53] Moreover, enhancer molecules that increase the readthrough activity of aminoglycosides and eRF3 degrader compounds have been shown to restore protein function from *TP53* and *PTEN* nonsense variants.[24] In this study, we show that combining aminoglycosides with newly discovered enhancer compounds can rescue protein function from two other tumor suppression genes, *NF1* and *CFTR*. While *NF1* is a well-characterized tumor suppressor gene that functions to downregulate GTP-Ras signal transduction pathways,[49,50] the tumor suppression function of CFTR, which plays a tumor suppressor role in colorectal cancer,[30,31] is much less understood.

CF was one of the first disease models for which aminoglycosides were shown to rescue significant protein expression and function from nonsense alleles in mammalian cells.[11] Moreover, clinical trials showed that topical nasal administration of gentamicin rescued CFTR function in the nasal epithelium of CF patients.[54,55] However, these studies were unable to show that the level of CFTR function needed for therapeutic improvements in CF patients (30%–35% of normal) could be reached.[56] Furthermore, ototoxicity[57] and nephrotoxicity[58] are often associated with long-term use of aminoglycosides. Consequently, the use of aminoglycosides to promote PTC readthrough has not yet been successfully implemented in the clinic.

Here, we show that a low EC_{10} dose of G418 paired with an enhancer is more effective at restoring CFTR function than G418 alone at its EC_{50}. Under the same culture conditions and following the TECC assay protocol for 16HBE cells, the 16HBE cells expressing wild-type CFTR show an average AUC of 2,500, with a baseline resistance value (Rt) around 250.[2] An AUC of 20%–30% of WT levels is considered the threshold for CFTR improvement in clinical candidates. The AUC value of 150 in Figures 2 and 3 is ∼6% of the WT level. Additionally, at the G418 EC_{10} concentration, the addition of CFTR correctors did not significantly enhance CFTR function above that of the G418 plus SRI-45987 without correctors, signifying that the CFTR protein translated by cells treated with the G418/enhancer combination was, for the most part, properly localized and functional. While we could not consistently observe restoration of full-length CFTR by immunoblotting, these data highlight the benefits of using a combinatory approach toward therapeutic rescue of CFTR. Furthermore, the addition of CFTR modulators, which were previously shown to enhance CFTR activity rescued via readthrough from several *CFTR* PTCs,[2,5,37,59,60] may further increase CFTR activity for other *CFTR* nonsense alleles.

Aminoglycoside antibiotics, the first compounds found to suppress translation termination at PTCs in eukaryotic cells,[61–63] remain some of the most effective compounds based on overall readthrough efficacy. However, the potency of these compounds is poor; thus, the high doses required for efficient readthrough frequently result in off-target effects that cause nephrotoxicity and ototoxicity.[13] The development of new synthetic aminoglycosides such as ELX-02, which was designed primarily to reduce off-target effects, exhibit reduced toxicity in mammalian cells compared to traditional aminoglycosides.[17,18,64] While the results of clinical trials assessing the ability of ELX-02 to treat Alport syndrome (ClinicalTrials.gov NCT05448755) and nephropathic cystinosis (ClinicalTrials.gov NCT04069260 have not yet been reported, clinical trials testing the ability of ELX-02 to attenuate endpoints associated with CF failed.[19] This could be due to the relatively high level of CFTR activity required to improve relevant pulmonary endpoints (∼30% of WT).[56]

While CF is a well-established disease model for therapeutic drug development, NF1 is less characterized, with much still unknown about neurofibromin regulation, endpoints, or the threshold of neurofibromin function required to provide therapeutic benefits in NF1 patients. Because NF1 is a common neurological disorder and 20% of *NF1* gene lesions result in in-frame PTCs, NF1 has the potential to serve as a robust model for the development of drugs that suppress nonsense mutations.[34] In the current study, we found that the aminoglycoside ELX-02 alone did not restore enough full-length neurofibromin protein to be detected via western blotting. Although ELX-02 did have a slight but significant effect on the level of Ras-GTP, *p*-ERK levels remained unchanged. This may be due to technical limitations or to the fact that ERK phosphorylation is more distal to direct neurofibromin function, leading to attenuation of the transduction signal, which is highly regulated and integrated with other signaling pathways.[65–67] However, combining ELX-02 with the aminoglycoside enhancer SRI-46124 resulted in reproducible detection of full-length neurofibromin (4%–5% of WT). The full-length neurofibromin rescued by PTC suppression was functional as evidenced by significant reductions in both Ras-GTP and *p*-ERK. When taken together, these results show that restoration of just 4%–5% of full-length neurofibromin can mediate a ∼50% functional output based on Ras-GTP and *p*-ERK/total-ERK levels in Schwann cells. Depending on the amino acid inserted at the PTC during readthrough, some of the restored neurofibromin may have only partial function. Based on this reasoning, the amount of functional neurofibromin restored could be even lower than 4% of WT. Despite NF1 having an autosomal dominant inheritance pattern, a

low threshold of neurofibromin restoration by nonsense suppression may be sufficient to rescue NF1 phenotypes. Further work is necessary to determine whether the low threshold for correction is similar in other cell types, such as fibroblasts or neurons. Since NF1 phenotypes are observed in multiple tissues, it will also be important to determine whether cell and tissue types respond differently to readthrough strategies *in vivo*. In addition, combining recent MEK inhibitors developed for NF1[68] with readthrough strategies may be a way to further attenuate NF1 phenotypes in multiple tissues.

By characterizing the effect of enhancers on aminoglycoside-mediated readthrough in different PTC contexts and in different *in vitro* disease model systems, our data lend strong support to the idea that enhancers can increase the potency of aminoglycosides for readthrough, which could allow a therapeutic threshold to be reached at much lower drug concentrations and help to ensure that patient safety is maintained. By enhancing the potency of aminoglycosides, readthrough enhancers could potentially permit current and/or developing readthrough agents to be used as effective, long-term therapies for many genetic disorders caused by nonsense mutations. If successfully implemented, these therapeutic strategies have the potential to treat millions of patients globally who carry a nonsense allele. Significantly, previous studies have indicated that the degree to which readthrough of normal stop codons occurs in cells treated with aminoglycosides and other readthrough agents is low.[2,69] This is likely due to differences in the ribonucleoprotein (RNP) structure formed at a normal stop codon versus a PTC.[7] In addition, interactions between the termination complex and factors bound to the 3′ UTR are also thought to promote more efficient termination at normal stop codons, making them less susceptible to readthrough.[7]

Other studies have also identified small molecules that function as aminoglycoside enhancers.[20,25–28] These compounds appear to have little readthrough effect alone but when co-administered with aminoglycosides, enhance their ability to promote readthrough compared to aminoglycoside monotherapy. For most enhancers, the mechanism has yet to be determined. However, one study found that the aminoglycoside enhancer, Y-320, upregulates CXC chemokine expression and ribosome biogenesis, suggesting that increased translation enhances the rescue of protein generated by readthrough.[26] Other potential enhancer mechanisms include increasing the effective cellular aminoglycoside concentration by either increasing their entry or by decreasing their efflux or metabolism, both mechanisms having the potential to directly affect aminoglycoside potency. Alternatively, enhancers could potentially modify cell signaling pathways that affect PTC suppression directly. Additional studies are needed to determine the mechanism of the enhancer compounds that were identified in this study.

MATERIALS AND METHODS

Tissue culture

HEK293 cells were obtained from ATCC (CRL-1573), gene-edited 16HBE14o- (16HBEge) cell lines were obtained from the Cystic Fibrosis Foundation Therapeutics Lab,[35] and patient-derived, immortalized Schwann cell lines ipn02.8 (*NF1* WT) and ipNF95.11b C (*NF1* null),[44] as well as i28cNF (*NF1* S1053X/S1078X)[70] were provided by Peggy Wallace, U. Florida. HEK293 and Schwann cells were cultured at 37°C with 6.5% CO_2 in Dulbecco's modified Eagle media (DMEM) (Thermo Fisher Scientific, MT10013CV) supplemented with 10% fetal bovine serum (FBS; Biotechne, S11550) and 1% minimum essential medium (MEM) non-essential amino acids (Thermo Fisher Scientific, MT25025CI). Additional details regarding optimizing seeding density for Schwann cells are shown in Figure S1. 16HBEge cell lines were cultured at 37°C with 5% CO2 in MEM (Gibco: 11095-080) supplemented with 10% FBS (Life Tech: A56707) and 1% penicillin+streptomycin (Thermo Fisher Scientific, Cat#15070-063).

Generation of readthrough reporter cell lines

NanoLuc RT/NMD high-throughput screening reporter

The NanoLuc-based RT/NMD reporter plasmid (pDB1362) (Figure 1A) previously described by Sharma et al. (2021)[2] was transfected into wild-type parental 16HBEge cells using Lipofectamine 2000 (Invitrogen-Thermo Fisher Scientific, Cat#11668-019). Stable transfectants were selected with 40 μg/mL zeocin (Thermo Fisher Scientific, Cat#R25005), and single-cell clones were expanded to generate monoclonal cell lines. Clone #15 was selected for high-throughput screening.

Halo-tag-hCFTR-5x5 G542X NanoLuc RT/NMD reporter

A NanoLuc RT/NMD reporter was generated that contains the human *CFTR* G542X context (Figure 1B). A gene block (gBlocks) was synthesized (Integrated DNA Technologies) that contains the Halo-tag, human *CFTR* G542X context (G542X codon situated between 5 codons of upstream and downstream *CFTR* context), and 50–60 nucleotides of homologous sequence located on each side of the Nhe I site in plasmid pDB1362.[2] NEBuilder HIFI DNA Assembly Master Mix (NEB Cat#2621) was used to insert the gBlocks into the Nhe I site of pDB1362. This plasmid was transfected into wild-type 16HBE14o- cells using Lipofectamine 2000 (Invitrogen-Thermo Fisher Scientific, Cat#11668-019). Cells stably expressing the reporter were selected with 40 μg/mL zeocin (Thermo Fisher Scientific, Cat#R25005), and single-cell clones were expanded to generate monoclonal cell lines. Clone #10 was used to validate HTS hits.

NF1 R816X dual luciferase readthough reporter

Dual luciferase readthrough reporters were constructed that carry the human *NF1* R816X PTC flanked by 3 codons of upstream and downstream natural human *NF1* sequence (Figure 4A). Primers DB4845: 5′-tcgacg gttaag aggtga atgtcc catg-3′ and DB4846: 5′-gatcca tggac attcac ctctta accg-3′ were annealed and cloned into the AscI/Sbf1 sites of plasmid pDB1497.[41] The plasmid was transfected into the wild-type Schwann cell line ipn02.8 using Lipofectamine LTX with PLUS reagent (Thermo Fisher Scientific, Cat#15338100). Cells stably expressing the reporter were selected using 50 μg/mL zeocin (Thermo Fisher Scientific, Cat#R25005), and single cell clones

were expanded to generate monoclonal stable lines. Clone #30 was used for readthrough assays.

High-throughput screening

A screen of 532,062 diverse compounds was performed using a single 30 μM concentration for each compound alone and in the presence of G418 EC_{20} (15 μg/mL) in 16HBEge reporter cells. Monoclonal 16HBEge reporter cells expressing the NanoLuc reporter (clone #15)[2] (Figure 1A) were cultured at 37°C with 5% CO_2 in Corning MEM, supplemented with 10% heat-inactivated FBS, 1% pen/strep, 1% HEPES, and 40 μg/mL zeocin. Cells were harvested and suspended in assay medium (Corning MEM supplemented with 10% heat-inactivated FBS, 1% Pen/Strep, and 1% HEPES), and 10,000 cells/well were seeded into Corning B370BC 384-well plates with a final assay volume of 30 μL. Testing and control compounds were diluted in assay medium at 6X concentration before addition to the assay plate in a 5 μL volume. Vehicle alone and G418 (100 μg/mL) served as controls; all assay conditions were run with a final concentration of 0.3% DMSO. 48 h after treatment, 30 μL of Promega NanoGlo reagent diluted 1:50 were then added to each well. After 10 min, plates were read on a BMG PheraStar reader in luminescence mode with the gain set to 2,500. Plate barcode-labeled raw data files were imported into the ActivityBase data management system using the XE module analysis template. Compound data were normalized to the average controls on the container plate. Percent activation versus log of the compound concentration was plotted and fit to a four-parameter logistic equation. Counter-screening of hits included running the same ± G418 (15 μg/mL) dose-response format with a cytotoxicity assay using ATP content as a readout (Promega CellTiterGlo).

Luciferase assays

Dual luciferase

Cells were seeded into 96-well plates, cultured without zeocin for 24 h, and then treated with vehicle alone or readthrough compound ± enhancers. 24 h later, cells were lysed with passive lysis buffer (Promega, E1960). Firefly and *Renilla* activities were assayed using the Promega Dual-Luciferase Reporter Assay System (Promega, E1960), with luminescence units measured by a Promega GloMax Discover Microplate Reader. Assays were performed in quadruplicate, the firefly (FF) relative light units (RLUs) normalized to the *Renilla* (Ren) ×100 (FF/Ren x100) were averaged, and the standard deviation (SD) was calculated. If a single outlier (>2.7 SD from average) was observed, it was removed; if multiple outliers were observed, the test treatment was repeated.

NanoLuc

Cells were seeded into 96-well plates without zeocin for 24 hours, treated with a readthrough compound or vehicle for an additional 48 hours, and then lysed in 20 μL Passive Lysis Buffer (PLB). Cell lysate (5 μL) from each well was used to perform the NanoLuc assay with the Nano-Glo luciferase assay system (Promega, Cat#N1120) using the GloMax *Discover* System (Promega). The lysate protein concentration was measured with the Bio-Rad Protein Assay Dye Reagent Concentrate (Bio-Rad, Cat#5000006). NanoLuc RLUs were normalized to total protein in each lysate (RLU/μg protein). The data are expressed as the mean (SD) of 3–4 replicates.

16HBEge TECC measurements

16HBEge cells carrying the *CFTR* G542X mutation were seeded onto HTS-24 transwell plate inserts (Corning: 3378) and cultured for 4 days at 37°C with 5% CO_2 in MEM (Gibco: 11095-080) supplemented with 10% FBS (Life Tech: A56707) and 1% penicillin-streptomycin (Thermo Fisher Scientific, Cat#15070-063). Prior to assay, vehicle (DMSO), G418 (EC_{10} = 6.53 μM or EC_{50} = 39.2 μM; Thermo Fisher Scientific: 10131035), a mixture of elexacaftor (E) (VX-445, 3 μM; Selleckchem: S8851), and tezacaftor (T) (VX-661, 3 μM; Selleckchem: S7059) (ET), or SRI-49587 (15 μM) alone or in various combinations were added to fresh media on both the basolateral and apical sides and incubated for 72 h. For equivalent current (Ieq) measurements, a chloride ion gradient was established from the basolateral to the apical side and performed as described previously.[2] Ieq changes were measured using a 24-channel voltage clamp (TECC-24; EP Design, Belgium) mounted on a robotic platform (Yamaha, Japan). After the addition of the cAMP agonist forskolin (10 μM; Cayman Chemical: NC1900942), ivacaftor (VX-770, 1 μM; Selleckchem: S1144), and CFTR Inh-172 (20 μM; Selleckchem: S7139), the AUC between the addition of forskolin and CFTR Inh-172 was used to determine the functional expression and rescue of CFTR.

Western blot analysis

Cells were seeded into 6-well plates, and after culturing for 24 h, cells were treated with different compounds as indicated for 72 h. Cells were lysed in M-PER Protein Expression Reagent (PI78501) supplemented with 5x cOmplete protease inhibitor tablets (Sigma-Aldrich, 11873580001), 5 mM EDTA, and 1 mM EGTA. The total lysate protein concentration was determined using the Bio-Rad Protein Assay (Bio-Rad, 5000006). For neurofibromin western blot analysis, 40 μg of protein from mutant ($NF1^{PTC}$ or $NF1^{null}$) or 2 μg protein from $NF1^{WT}$ lysates was subjected to SDS-PAGE, unless otherwise stated. The lower amount of $NF1^{WT}$ protein was used to ensure direct correlation with restored neurofibromin levels. For all other western blot analyses, 20 μg of protein was used per sample. Proteins were then transferred to Immobilon-FL PVDF membrane (Thermo Fisher Scientific, IPFL00010 or IPFL85R), blocked in 5% dried milk dissolved in TPBS, and then incubated with primary antibodies diluted as follows: 1:500 mouse anti-GAPDH (DSHB, hGAPDH-2G7), 1:500 rabbit anti-pERK1/2 (Abcam, ab223500), 1:1,000 rabbit anti-ERK1/2 (Abcam, ab184699), 1:1,000 rabbit anti-NF1 (Abcam, ab238142), 1:1,000 rabbit anti-eRF1 (Thermo Fisher Scientific, PIPA528777), or 1:1,000 rabbit anti-eRF3 (Thermo Fisher Scientific, PIPA528256) at room temperature for 2 h. Blots were then washed and incubated with secondary antibodies 1:20,000 Li-Cor IRDye 680 RD goat anti-rabbit (Thermo Fisher Scientific, NC0252291) and Li-Cor IRDye 800 CW goat anti-mouse (Thermo Fisher Scientific, NC9401841). Blots were exposed on a Li-Cor Odyssey Clx Infrared Fluorescent

Western Scanning System. Images were analyzed using ImageStudioLite software (Li-Cor).

Ras-GTP assay

Ras-GTP assays were performed using the Ras G-LISA Activation Assay Kit (Cytoskeleton, Inc, BK131). Schwann cells were treated with readthrough compounds or vehicle as described for western blotting previously; half the samples were also treated with 50 ng/mL human EGF (epidermal growth factor; Thermo Fisher Scientific, PHG0311) at 37°C for 5 min to activate Ras. Cells were then lysed in 100 μL of the kit cell lysis buffer; 10 μL of lysate was used to quantitate protein levels. For assay, the samples were diluted to equal protein concentrations; 90 μL of each was added to 90 μL of binding buffer on ice. 50 μL of each sample was added to each well of the kit 96-well plate and processed according to manufacturer instructions. Luminescence was measured on a Promega GloMax Discover Microplate Reader; the mean (SD) luminescence for each cohort was calculated.

Quantitative reverse-transcription PCR

Cells were treated as described for the western blot experiments. Total RNA was isolated from cells using the Qiagen RNeasy Mini Kit (Qiagen, 74106) with optional QIAshredder (Qiagen, 79656). 1 μg of RNA was reverse transcribed using BioRad iScript reverse transcriptase (Bio-Rad, 1708841) in a Bio-Rad C1000 Touch Thermal Cycler. 2 μL of the cDNA served as the template for qPCR using iQ SYBR Green Supermix (Bio-Rad, 1708882) and performed with a BioRad CFX96 Real-Time System. qPCR primers are shown in Table S1.

Statistical analysis

All statistical analyses were performed using Prism9 software. A *t* test, ordinary one-way ANOVA or ordinary one-way ANOVA followed by Holm-Sidak's test were used to determine *p* values and whether significant differences between control and experimental samples were observed. A post-hoc Dunnett test was performed to correct for multiple comparisons.

DATA AND CODE AVAILABILITY

All data are shown. Raw data will be available in the supplemental information or from the corresponding author upon request.

ACKNOWLEDGMENTS

This project was supported by the National Institutes of Health (2P30DK072482-14), the Cystic Fibrosis Foundation (subcontract 858345; DAVIS24R0), and the Gilbert Family Foundation (523001). We also wish to acknowledge Lynn Rasmussen who helped optimize the initial high-throughput screening platform at Southern Research that was used to identify readthrough enhancer compounds.

AUTHOR CONTRIBUTIONS

Conceptualization was performed by C.A.-S., K.M.K., D.W., R.A.K., S.M.R., and D.M.B. Data were curated by J.S., J.C., K.T., L.F., M.D., J.R.B., P.V., O.M.-C., and C.A.-S. Formal analysis was carried out by J.S., J.C., L.F., M.D., J.R.B., P.V., O.M.-C., and C.A.-S. Funding was acquisitioned by C.A.-S., K.M.K., D.W., R.A.K., S.M.R., and D.M.B. Investigation was performed by J.S., J.C., K.T., L.F., M.D., and H.W., while methodology was performed by J.S., J.C., L.F., H.W., O.M.-C., C.A.-S., and R.A.K. Project administration consisted of C.A.-S., K.M.K., D.W., R.A.K., S.M.R., and D.M.B. Resources were obtained and maintained by J.C., K.T., L.F., M.D., H.W., J.R.B., P.V., C.A.-S., D.W., R.A.K., S.M.R., and D.M.B. R.B., P.V., C.A.S., K.M.K., D.W., R.A.K., S.M.R., and D.M.B. supervised project progress. J.S., J.C., K.T., L.F., M.D., J.R.B., P.V., and O.M.-C. performed validation while J.S., J.C., K.T., L.F., M.D., P.V., and C.A.-S. managed visualization. The original draft was written by J.S., K.M.K., and D.M.B., while J.S., J.C., L.F., K.M.K., and D.M.B. were involved in review and editing.

DECLARATION OF INTERESTS

None of the authors have any financial conflicts of interest to declare.

SUPPLEMENTAL INFORMATION

Supplemental information can be found online at https://doi.org/10.1016/j.omton.2026.201173.

REFERENCES

1. Mort, M., Ivanov, D., Cooper, D.N., and Chuzhanova, N.A. (2008). A meta-analysis of nonsense mutations causing human genetic disease. Hum. Mutat. *29*, 1037–1047.
2. Sharma, J., Du, M., Wong, E., Mutyam, V., Li, Y., Chen, J., Wangen, J., Thrasher, K., Fu, L., Peng, N., et al. (2021). A small molecule that induces translational readthrough of CFTR nonsense mutations by eRF1 depletion. Nat. Commun. *12*, 4358. https://doi.org/10.1038/s41467-021-24575-x.
3. Keeling, K.M., Wang, D., Dai, Y., Murugesan, S., Chenna, B., Clark, J., Belakhov, V., Kandasamy, J., Velu, S.E., Baasov, T., and Bedwell, D.M. (2013). Attenuation of nonsense-mediated mRNA decay enhances in vivo nonsense suppression. PLoS One *8*, e60478. https://doi.org/10.1371/journal.pone.0060478.
4. Keeling, K.M., Lanier, J., Du, M., Salas-Marco, J., Gao, L., Kaenjak-Angeletti, A., and Bedwell, D.M. (2004). Leaky termination at premature stop codons antagonizes nonsense-mediated mRNA decay in *S. cerevisiae*. RNA *10*, 691–703.
5. Lee, R.E., Lewis, C.A., He, L., Bulik-Sullivan, E.C., Gallant, S.C., Mascenik, T.M., Dang, H., Cholon, D.M., Gentzsch, M., Morton, L.C., et al. (2022). Small molecule eRF3a degraders rescue CFTR nonsense mutations by promoting premature termination codon readthrough. J. Clin. Investig. *132*, e154571. https://doi.org/10.1172/JCI154571.
6. Alkalaeva, E.Z., Pisarev, A.V., Frolova, L.Y., Kisselev, L.L., and Pestova, T.V. (2006). In Vitro Reconstitution of Eukaryotic Translation Reveals Cooperativity between Release Factors eRF1 and eRF3. Cell *125*, 1125–1136.
7. Wu, C., Roy, B., He, F., Yan, K., and Jacobson, A. (2020). Poly(A)-Binding Protein Regulates the Efficiency of Translation Termination. Cell Rep. *33*, 108399. https://doi.org/10.1016/j.celrep.2020.108399.
8. Lee, H.L.R., and Dougherty, J.P. (2012). Pharmaceutical therapies to recode nonsense mutations in inherited diseases. Pharmacol. Ther. *136*, 227–266. https://doi.org/10.1016/j.pharmthera.2012.07.007.
9. Spelier, S., van Doorn, E.P.M., van der Ent, C.K., Beekman, J.M., and Koppens, M.A.J. (2023). Readthrough compounds for nonsense mutations: bridging the translational gap. Trends Mol. Med. *29*, 297–314. https://doi.org/10.1016/j.molmed.2023.01.004.
10. Temaj, G., Telkoparan-Akillilar, P., Nuhii, N., Chichiarelli, S., Saha, S., and Saso, L. (2023). Recoding of Nonsense Mutation as a Pharmacological Strategy. Biomedicines *11*, 659. https://doi.org/10.3390/biomedicines11030659.
11. Howard, M., Frizzell, R.A., and Bedwell, D.M. (1996). Aminoglycoside antibiotics restore CFTR function by overcoming premature stop mutations. Nat. Med. *2*, 467–469.
12. Bedwell, D.M., Kaenjak, A., Benos, D.J., Bebok, Z., Bubien, J.K., Hong, J., Tousson, A., Clancy, J.P., and Sorscher, E.J. (1997). Suppression of a CFTR premature stop mutation in a bronchial epithelial cell line. Nat. Med. *3*, 1280–1284.
13. Begg, E.J., and Barclay, M.L. (1995). Aminoglycosides–50 years on. Br. J. Clin. Pharmacol. *39*, 597–603.
14. Guchhait, S., Khononov, A., Pieńko, T., Belakhov, V., and Baasov, T. (2023). Balancing Nonsense Mutation Readthrough and Toxicity of Designer Aminoglycosides for Treatment of Genetic Diseases. ACS Med. Chem. Lett. *14*, 794–801. https://doi.org/10.1021/acsmedchemlett.3c00089.

15. Shulman, E., Belakhov, V., Wei, G., Kendall, A., Meyron-Holtz, E.G., Ben-Shachar, D., Schacht, J., and Baasov, T. (2014). Designer Aminoglycosides That Selectively Inhibit Cytoplasmic Rather than Mitochondrial Ribosomes Show Decreased Ototoxicity: A STRATEGY FOR THE TREATMENT OF GENETIC DISEASES. J. Biol. Chem. *289*, 2318–2330. https://doi.org/10.1074/jbc.M113.533588.

16. Nudelman, I., Rebibo-Sabbah, A., Shallom-Shezifi, D., Hainrichson, M., Stahl, I., Ben-Yosef, T., and Baasov, T. (2006). Redesign of aminoglycosides for treatment of human genetic diseases caused by premature stop mutations. Bioorg. Med. Chem. Lett. *16*, 6310–6315.

17. Leubitz, A., Vanhoutte, F., Hu, M.Y., Porter, K., Gordon, E., Tencer, K., Campbell, K., Banks, K., and Haverty, T. (2021). A Randomized, Double-Blind, Placebo-Controlled, Multiple Dose Escalation Study to Evaluate the Safety and Pharmacokinetics of ELX-02 in Healthy Subjects. Clin. Pharmacol. Drug Dev. *10*, 859–869. https://doi.org/10.1002/cpdd.914.

18. Leubitz, A., Frydman-Marom, A., Sharpe, N., van Duzer, J., Campbell, K.C.M., and Vanhoutte, F. (2019). Safety, Tolerability, and Pharmacokinetics of Single Ascending Doses of ELX-02, a Potential Treatment for Genetic Disorders Caused by Nonsense Mutations, in Healthy Volunteers. Clin. Pharmacol. Drug Dev. *8*, 984–994. https://doi.org/10.1002/cpdd.647.

19. Eloxx Pharmaceuticals, I. (2023). Eloxx Pharmaceuticals Announces Final Data Assessment from Phase 2 Combination Clinical Trial of ELX-02 in Class 1 Cystic Fibrosis (CF) Patients. Eloxx Pharmaceuticals Announces Final Data. https://www.biospace.com/eloxx-pharmaceuticals-announces-final-data-assessment-from-phase-2-combination-clinical-trial-of-elx-02-in-class-1-cystic-fibrosis-cf-patients.

20. Du, M., Keeling, K.M., Fan, L., Liu, X., and Bedwell, D.M. (2009). Poly-L-aspartic Acid Enhances and Prolongs Gentamicin-mediated Suppression of the CFTR-G542X Mutation in a Cystic Fibrosis Mouse Model. J. Biol. Chem. *284*, 6885–6892. https://doi.org/10.1074/jbc.M806728200.

21. Usuki, F., Yamashita, A., Shiraishi, T., Shiga, A., Onodera, O., Higuchi, I., and Ohno, S. (2013). Inhibition of SMG-8, a subunit of SMG-1 kinase, ameliorates nonsense-mediated mRNA decay-exacerbated mutant phenotypes without cytotoxicity. Proc. Natl. Acad. Sci. USA *110*, 15037–15042. https://doi.org/10.1073/pnas.1300654110.

22. Xue, X., Mutyam, V., Tang, L., Biswas, S., Du, M., Jackson, L.A., Dai, Y., Belakhov, V., Shalev, M., Chen, F., et al. (2014). Synthetic aminoglycosides efficiently suppress cystic fibrosis transmembrane conductance regulator nonsense mutations and are enhanced by ivacaftor. Am. J. Respir. Cell Mol. Biol. *50*, 805–816. https://doi.org/10.1165/rcmb.2013-0282OC.

23. Zhang, M., Heldin, A., Palomar-Siles, M., Öhlin, S., Bykov, V.J.N., and Wiman, K.G. (2017). Synergistic Rescue of Nonsense Mutant Tumor Suppressor p53 by Combination Treatment with Aminoglycosides and Mdm2 Inhibitors. Front. Oncol. *7*, 323. https://doi.org/10.3389/fonc.2017.00323.

24. Heldin, A., Cancer, M., Palomar-Siles, M., Öhlin, S., Zhang, M., Sun-Zhang, A., Mariani, A., Liu, J., Bykov, V.J.N., and Wiman, K.G. (2023). Novel compounds that synergize with aminoglycoside G418 or eRF3 degraders for translational readthrough of nonsense mutant. RNA Biol. *20*, 368–383. https://doi.org/10.1080/15476286.2023.2222250.

25. Baradaran-Heravi, A., Balgi, A.D., Zimmerman, C., Choi, K., Shidmoossavee, F.S., Tan, J.S., Bergeaud, C., Krause, A., Flibotte, S., Shimizu, Y., et al. (2016). Novel small molecules potentiate premature termination codon readthrough by aminoglycosides. Nucleic Acids Res. *44*, 6583–6598. https://doi.org/10.1093/nar/gkw638.

26. Hosseini-Farahabadi, S., Baradaran-Heravi, A., Zimmerman, C., Choi, K., Flibotte, S., and Roberge, M. (2021). Small molecule Y-320 stimulates ribosome biogenesis, protein synthesis, and aminoglycoside-induced premature termination codon readthrough. PLoS Biol. *19*, e3001221. https://doi.org/10.1371/journal.pbio.3001221.

27. Rabea, S.M., Baradaran-Heravi, A., Balgi, A.D., Krause, A., Hosseini Farahabadi, S., Roberge, M., and Grierson, D.S. (2019). 2-Aminothiazole-4-carboxamides Enhance Readthrough of Premature Termination Codons by Aminoglycosides. ACS Med. Chem. Lett. *10*, 726–731. https://doi.org/10.1021/acsmedchemlett.8b00610.

28. Wong, K.M., Wegener, E., Baradaran-Heravi, A., Huppke, B., Gärtner, J., and Huppke, P. (2023). Evaluation of Novel Enhancer Compounds in Gentamicin-Mediated Readthrough of Nonsense Mutations in Rett Syndrome. Int. J. Mol. Sci. *24*, 11665. https://doi.org/10.3390/ijms241411665.

29. Cutting, G.R. (2015). Cystic fibrosis genetics: from molecular understanding to clinical application. Nat. Rev. Genet. *16*, 45–56. https://doi.org/10.1038/nrg3849.

30. Than, B.L.N., Linnekamp, J.F., Starr, T.K., Largaespada, D.A., Rod, A., Zhang, Y., Bruner, V., Abrahante, J., Schumann, A., Luczak, T., et al. (2016). CFTR is a tumor suppressor gene in murine and human intestinal cancer. Oncogene *35*, 4179–4187. https://doi.org/10.1038/onc.2015.483.

31. Liu, C., Song, C., Li, J., and Sun, Q. (2020). CFTR Functions as a Tumor Suppressor and Is Regulated by DNA Methylation in Colorectal Cancer. Cancer Manag. Res. *12*, 4261–4270. https://doi.org/10.2147/CMAR.S248539.

32. Sosnay, P., Salinas, D., White, T., Ren, C., Farrell, P., Raraigh, K., Girodon, E., and Castellani, C. (2017). Applying cystic fibrosis transmembrane conductance genetics and CFTR2 data to facilitate diagnoses. J. Peidatri. *181S*, S27–S32.e1. https://doi.org/10.1016/j.jpeds.2016.09.063.

33. Legius, E., Messiaen, L., Wolkenstein, P., Pancza, P., Avery, R.A., Berman, Y., Blakeley, J., Babovic-Vuksanovic, D., Cunha, K.S., Ferner, R., et al. (2021). Revised diagnostic criteria for neurofibromatosis type 1 and Legius syndrome: an international consensus recommendation. Genet. Med. *23*, 1506–1513. https://doi.org/10.1038/s41436-021-01170-5.

34. Messiaen, L.M., and Wimmer, K. (2008). NF1 mutational spectrum. In Mo*nographs in Human Genetics:* Neurofibromatoses, K. D, ed. (Karger), pp. 63–77. https://doi.org/10.1159/000126545.

35. Valley, H.C., Bukis, K.M., Bell, A., Cheng, Y., Wong, E., Jordan, N.J., Allaire, N.E., Sivachenko, A., Liang, F., Bihler, H., et al. (2019). Isogenic cell models of cystic fibrosis-causing variants in natively expressing pulmonary epithelial cells. J. Cyst. Fibros. *18*, 476–483. https://doi.org/10.1016/j.jcf.2018.12.001.

36. Thrasher, K., Chen, J., Fu, L., Benson, D., Ekstrom, J., Hargett, A.A., Renfrow, M.B., Rowe, S.M., Keeling, K.M., and Bedwell, D.M. (2026). Identity, functional consequences, and context effects of amino acids inserted during suppression of CFTR nonsense mutations. J. Cyst. Fibros. *25*, 106–117. https://doi.org/10.1016/j.jcf.2025.09.

37. Xue, X., Mutyam, V., Thakerar, A., Mobley, J., Bridges, R.J., Rowe, S.M., Keeling, K.M., and Bedwell, D.M. (2017). Identification of the Amino Acids Inserted During Suppression of CFTR Nonsense Mutations and Determination of Their Functional Consequences. Hum. Mol. Genet. *26*, 3116–3129. https://doi.org/10.1093/hmg/ddx196.

38. Roy, B., Friesen, W.J., Tomizawa, Y., Leszyk, J.D., Zhuo, J., Johnson, B., Dakka, J., Trotta, C.R., Xue, X., Mutyam, V., et al. (2016). Ataluren stimulates ribosomal selection of near-cognate tRNAs to promote nonsense suppression. Proc. Natl. Acad. Sci. USA *113*, 12508–12513. https://doi.org/10.1073/pnas.1605336113.

39. Heijerman, H.G.M., McKone, E.F., Downey, D.G., Van Braeckel, E., Rowe, S.M., Tullis, E., Mall, M.A., Welter, J.J., Ramsey, B.W., McKee, C.M., et al. (2019). Efficacy and safety of the elexacaftor plus tezacaftor plus ivacaftor combination regimen in people with cystic fibrosis homozygous for the F508del mutation: a double-blind, randomised, phase 3 trial. Lancet *394*, 1940–1948. https://doi.org/10.1016/S0140-6736(19)32597-8.

40. Riccardi, V.M. (1981). Von Recklinghausen neurofibromatosis. N. Engl. J. Med. *305*, 1617–1627. https://doi.org/10.1056/NEJM198112313052704.

41. Mangkalaphiban, K., Fu, L., Du, M., Thrasher, K., Keeling, K.M., Bedwell, D.M., and Jacobson, A. (2024). Extended stop codon context predicts nonsense codon readthrough efficiency in human cells. Nat. Commun. *15*, 2486. https://doi.org/10.1038/s41467-024-46703-z.

42. Lynch, S.R., and Puglisi, J.D. (2001). Structure of a eukaryotic decoding region A-site RNA. J. Mol. Biol. *306*, 1023–1035.

43. Yoshizawa, S., Fourmy, D., and Puglisi, J.D. (1998). Structural origins of gentamicin antibiotic action. EMBO J. *17*, 6437–6448.

44. Li, H., Chang, L.J., Neubauer, D.R., Muir, D.F., and Wallace, M.R. (2016). Immortalization of human normal and NF1 neurofibroma Schwann cells. Lab. Invest. *96*, 1105–1115. https://doi.org/10.1038/labinvest.2016.88.

45. Norton, K.K., Mahadeo, D.K., Geist, R.T., and Gutmann, D.H. (1996). Expression of the neurofibromatosis 1 (NF1) gene during growth arrest. Neuroreport *7*, 601–604. https://doi.org/10.1097/00001756-199601310-00054.

46. Cichowski, K., Santiago, S., Jardim, M., Johnson, B.W., and Jacks, T. (2003). Dynamic regulation of the Ras pathway via proteolysis of the NF1 tumor suppressor. Genes Dev. *17*, 449–454. https://doi.org/10.1101/gad.1054703.

47. Pros, E., Larriba, S., López, E., Ravella, A., Gili, M.L., Kruyer, H., Valls, J., Serra, E., and Lázaro, C. (2006). NF1 mutation rather than individual genetic variability is the main determinant of the NF1-transcriptional profile of mutations affecting splicing. Hum. Mutat. *27*, 1104–1114. https://doi.org/10.1002/humu.20396.

48. Ko, W., Porter, J.J., Sipple, M.T., Edwards, K.M., and Lueck, J.D. (2022). Efficient Suppression of Endogenous CFTR Nonsense Mutations Using Anticodon Engineered Transfer RNAs. Mol. Ther. Nucleic Acids *28*, 685–701. https://doi.org/10.1016/j.omtn.2022.04.033.

49. Xu, G.F., Lin, B., Tanaka, K., Dunn, D., Wood, D., Gesteland, R., White, R., Weiss, R., and Tamanoi, F. (1990). The catalytic domain of the neurofibromatosis type 1 gene product stimulates ras GTPase and complements ira mutants of S. cerevisiae. Cell *63*, 835–841. https://doi.org/10.1016/0092-8674(90)90149-9.

50. Martin, G.A., Viskochil, D., Bollag, G., McCabe, P.C., Crosier, W.J., Haubruck, H., Conroy, L., Clark, R., O'Connell, P., and Cawthon, R.M. (1990). The GAP-related domain of the neurofibromatosis type 1 gene product interacts with ras p21. Cell *63*, 843–849. https://doi.org/10.1016/0092-8674(90)90150-d.

51. Leevers, S.J., and Marshall, C.J. (1992). Activation of extracellular signal-regulated kinase, ERK2, by p21ras oncoprotein. EMBO J. *11*, 569–574. https://doi.org/10.1002/j.1460-2075.1992.tb05088.x.

52. Robbins, D.J., Cheng, M., Zhen, E., Vanderbilt, C.A., Feig, L.A., and Cobb, M.H. (1992). Evidence for a Ras-dependent extracellular signal-regulated protein kinase (ERK) cascade. Proc. Natl. Acad. Sci. USA *89*, 6924–6928. https://doi.org/10.1073/pnas.89.15.6924.

53. Torices, L., Nunes-Xavier, C.E., and Pulido, R. (2025). Therapeutic Potential of Translational Readthrough at Disease-Associated Premature Termination Codons From Tumor Suppressor Genes. IUBMB Life *77*, e70018. https://doi.org/10.1002/iub.70018.

54. Wilschanski, M., Yahav, Y., Yaacov, Y., Blau, H., Bentur, L., Rivlin, J., Aviram, M., Bdolah-Abram, T., Bebok, Z., Shushi, L., et al. (2003). Gentamicin-induced correction of CFTR function in patients with cystic fibrosis and CFTR stop mutations. N. Engl. J. Med. *349*, 1433–1441.

55. Wilschanski, M., Famini, C., Blau, H., Rivlin, J., Augarten, A., Avital, A., Kerem, B., and Kerem, E. (2000). A pilot study of the effect of gentamicin on nasal potential difference measurements in cystic fibrosis patients carrying stop mutations. Am. J. Respir. Crit. Care Med. *161*, 860–865.

56. Kerem, E. (2004). Pharmacologic therapy for stop mutations: how much CFTR activity is enough? Curr. Opin. Pulm. Med. *10*, 547–552.

57. Nakashima, T., Teranishi, M., Hibi, T., Kobayashi, M., and Umemura, M. (2000). Vestibular and cochlear toxicity of aminoglycosides–a review. Acta Otolaryngol. *120*, 904–911.

58. Nagai, J., and Takano, M. (2004). Molecular aspects of renal handling of aminoglycosides and strategies for preventing the nephrotoxicity. Drug Metab. Pharmacokinet. *19*, 159–170.

59. Chen, J., Thrasher, K., Fu, L., Wang, W., Aghamohammadzadeh, S., Wen, H., Tang, L., Keeling, K.M., Falk Libby, E., Bedwell, D.M., and Rowe, S.M. (2023). The synthetic aminoglycoside ELX-02 induces readthrough of G550X-CFTR producing superfunctional protein that can be further enhanced by CFTR modulators. Am. J. Physiol. Lung Cell. Mol. Physiol. *324*, L756–L770. https://doi.org/10.1152/ajplung.00038.2023.

60. Mutyam, V., Du, M., Xue, X., Keeling, K.M., White, E.L., Bostwick, J.R., Rasmussen, L., Liu, B., Mazur, M., Hong, J.S., et al. (2016). Discovery of Clinically Approved Agents That Promote Suppression of CFTR Nonsense Mutations. Am. J. Respir. Crit. Care Med. *194*, 1092–1103. https://doi.org/10.1164/rccm.201601-0154OC.

61. Singh, A., Ursic, D., and Davies, J. (1979). Phenotypic suppression and misreading *Saccharomyces cerevisiae*. Nature *277*, 146–148.

62. Palmer, E., Wilhelm, J.M., and Sherman, F. (1979). Phenotypic suppression of nonsense mutants in yeast by aminoglycoside antibiotics. Nature *277*, 148–150.

63. Burke, J.F., and Mogg, A.E. (1985). Suppression of a nonsense mutation in mammalian cells in vivo by the aminoglycoside antibiotics G-418 and paromomycin. Nucleic Acids Res. *13*, 6265–6272.

64. Bidou, L., Bugaud, O., Belakhov, V., Baasov, T., and Namy, O. (2017). Characterization of new-generation aminoglycoside promoting premature termination codon readthrough in cancer cells. RNA Biol. *14*, 378–388. https://doi.org/10.1080/15476286.2017.1285480.

65. Mendoza, M.C., Er, E.E., and Blenis, J. (2011). The Ras-ERK and PI3K-mTOR pathways: cross-talk and compensation. Trends Biochem. Sci. *36*, 320–328. https://doi.org/10.1016/j.tibs.2011.03.006.

66. Shen, Y.H., Godlewski, J., Zhu, J., Sathyanarayana, P., Leaner, V., Birrer, M.J., Rana, A., and Tzivion, G. (2003). Cross-talk between JNK/SAPK and ERK/MAPK pathways: sustained activation of JNK blocks ERK activation by mitogenic factors. J. Biol. Chem. *278*, 26715–26721. https://doi.org/10.1074/jbc.M303264200.

67. Biswas, S., Tikader, B., Kar, S., and Viswanathan, G.A. (2022). Modulation of signaling cross-talk between pJNK and pAKT generates optimal apoptotic response. PLoS Comput. Biol. *18*, e1010626. https://doi.org/10.1371/journal.pcbi.1010626.

68. Galvin, R., Watson, A.L., Largaespada, D.A., Ratner, N., Osum, S., and Moertel, C.L. (2021). Neurofibromatosis in the Era of Precision Medicine: Development of MEK Inhibitors and Recent Successes with Selumetinib. Curr. Oncol. Rep. *23*, 45. https://doi.org/10.1007/s11912-021-01032-y.

69. Wangen, J.R., and Green, R. (2020). Stop Codon Context Influences Genome-Wide Stimulation of Termination Codon Readthrough by Aminoglycosides. eLife *9*, 1–78. https://doi.org/10.1101/798579.

70. Li, H., Pemov, A., Allaway, R., Muir, D.F., Chang, L.J., Banerjee, J., Scott, A.J., Nagy, J.M.W., Liu, J., Carrió, M., et al. (2025). Immortalization and characterization of Schwann cell lines derived from NF1 associated cutaneous neurofibromas. PLoS One *21*, e0344782. https://doi.org/10.1371/journal.pone.0344792.

OMTON, Volume *34*

Supplemental information

Identification of small molecules that enhance aminoglycoside-mediated suppression of *CFTR* and *NF1* nonsense mutations

Joshua Sammons, Jianguo Chen, Kari Thrasher, Lianwu Fu, Ming Du, Hui Wen, J. Robert Bostwick, Paige Vinson, Omar Moukha-Chafiq, Corinne Augelli-Szafran, Kim M. Keeling, Deeann Wallis, Robert A. Kesterson, Steven M. Rowe, and David M. Bedwell

Table S1: qPCR Primers

Primer Name	Primer Sequence
hNF1 Forward	5'-GCTTTCGTATAAGCCCTCACAA-3'
hNF1 Reverse	5'-GCGGAATTGGTGATGATTCGATG -3'
hGUSb Forward	5'-CTGTACACGACACCCACCAC-3'
hGUSb Reverse	5'-ATTCGCCACGACTTTGTT-3'
hTBP Forward	5'-TGCACAGGAGCCAAGAGTGAA-3'
hTBP Reverse	5'-CACATCACAGCTCCCCACCA-3'

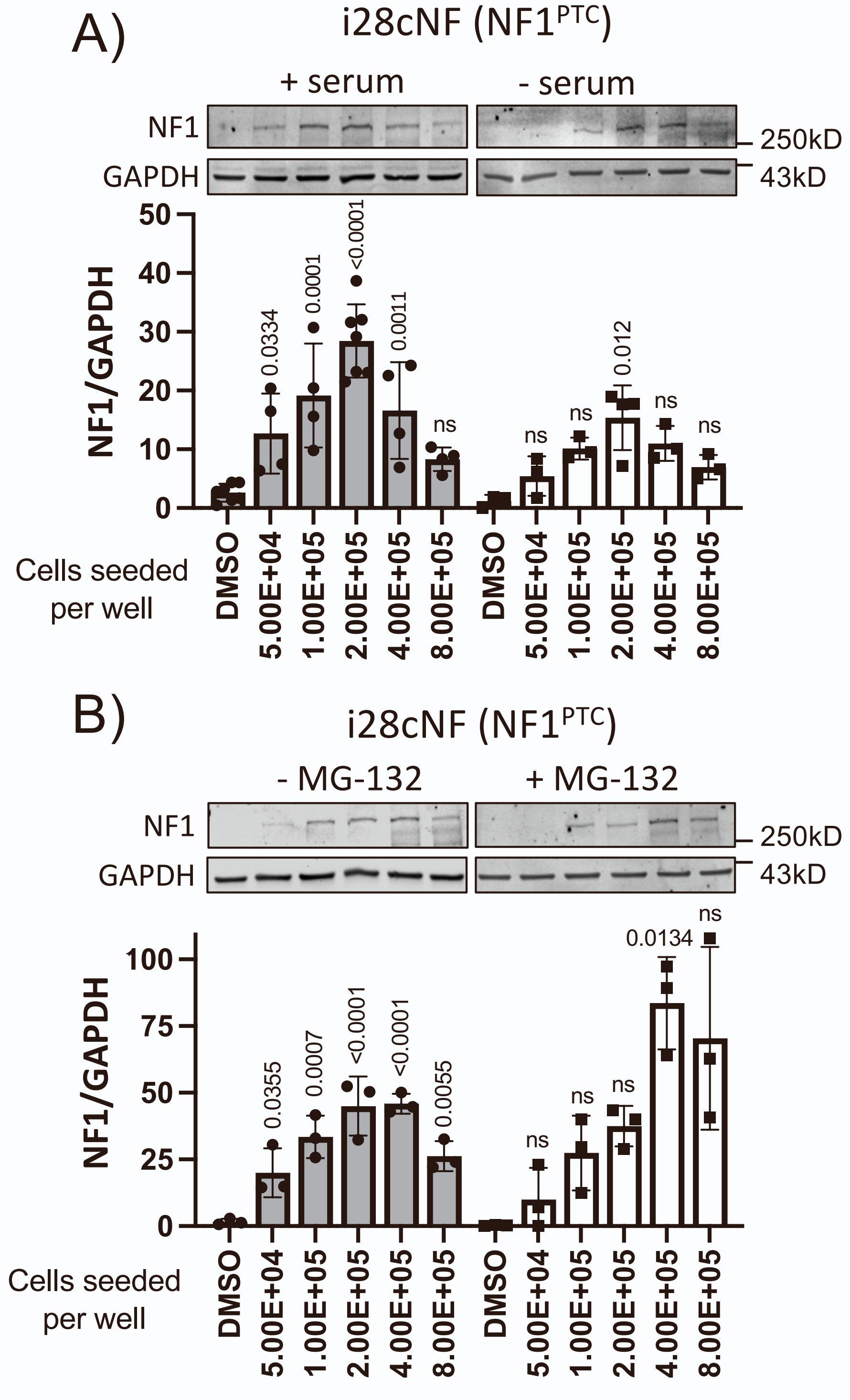

Figure S1: Optimization of neurofibromin expression in cultured NF1 Schwann cells under readthrough conditions. A) *NF1*^{PTC} (i28cNF) Schwann cells were seeded at different cell densities ($5x10^4$-$8x10^5$ cells per $9.5cm^2$ =1 well of a 6-well dish) and treated with 73.7 µM ELX-02 + 7.5 µM SRI-46124 +/- 10% FBS for 72 hours to determine the effect of confluency and serum on rescue of neurofibromin expression *via* readthrough. **B)** *NF1*^{PTC} (i28cNF) Schwann cells were seeded at different densities and treated with 73.7 µM ELX-02 + 7.5 µM SRI-46124 for 72 hours. Proteasomal inhibitor MG-132 was added during the last 24 hours to inhibit neurofibromin degradation. For A and B, DMSO treated cells were seeded at a $2x10^5$ cells per $9.5cm^2$ (1 well of a 6-well dish). For the “+ serum” and “- MG-132” cohorts, p-values (above columns) compare experimental cohorts to the respective vehicle (DMSO) control. For the “- serum” and ”+ MG-132” cohorts, p-values (above columns) compare experimental cohorts to the cells lacking serum or MG-132 at the same seeding density. Data are expressed as mean (SD) and were statistically analyzed using an ordinary one-way ANOVA followed by the Dunnett’s test. p values >0.05 are non-significant (ns).

Additional text related to Figure S1. Much is still not understood about how neurofibromin expression and function are regulated or how neurofibromin function intersects other signaling pathways. For example, the effect of various growth conditions on neurofibromin expression is not well-characterized. It was previously shown that multiple cell types under serum starvation express higher levels of neurofibromin than serum replete cells [1-3]. However, we report here that it can have the opposite effect on generation of full-length neurofibromin *via* PTC readthrough in Schwann cells, potentially by altering translation rates or by directly altering mechanisms related to readthrough. Further, our data suggest that sub-confluency of cultured cell cultures may be optimal for native neurofibromin expression as well as its rescue *via* readthrough. Once cells reach confluency, neurofibromin appears to be reduced in a proteasome-dependent manner.

Supplemental Material References:

1. Cichowski K, Santiago S, Jardim M, Johnson BW, Jacks T: **Dynamic regulation of the Ras pathway via proteolysis of the NF1 tumor suppressor.** *Genes Dev* **17:**449-454 (2003).
2. Gutmann DH, Cole JL, Collins FS: **Modulation of neurofibromatosis type 1 gene expression during in vitro myoblast differentiation.** *J Neurosci Res* **37:**398-405 (1994).
3. Norton KK, Mahadeo DK, Geist RT, Gutmann DH: **Expression of the neurofibromatosis 1 (NF1) gene during growth arrest.** *Neuroreport* **7:**601-604 (1996)
